# Nonverbal Rationality? 2‐Year‐Old Children, Dogs, and Pigs Show Unselective Responses to Unreliability but to Different Degrees

**DOI:** 10.1111/cdev.70020

**Published:** 2025-08-04

**Authors:** Kirsten H. Blakey, Eva Rafetseder, Giacomo Melis, Ariane Veit, Kea Amelung, Franziska Freudensprung, Kinga Kovacs, Zsófia Virányi

**Affiliations:** ^1^ Philosophy, Faculty of Arts and Humanities University of Stirling Stirling UK; ^2^ Psychology, Faculty of Natural Sciences University of Stirling Stirling UK; ^3^ Psychology, University of Toronto Toronto Ontario Canada; ^4^ Messerli Research Institute University of Veterinary Medicine, Medical University of Vienna, University of Vienna Vienna Austria; ^5^ University of Greifswald Greifswald Germany

**Keywords:** belief revision, rational thinking, reflection

## Abstract

Some philosophers argue that reflection is key to rational thinking. By tying reflective thinking to language, they struggle to account for minimally verbal infants and exclude nonhuman animals. This study assessed processing of undermining defeaters—a basic form of reflective thinking—in 36 two‐year‐old British children (13 female; *M*_age_ = 30.4 months, 98% White), 39 dogs (18 female), and 21 pigs (9 female), tested between 2022 and 2023. Informants acted on two screens: one informant reliably indicated a rewarded location; the other informant did not. Informants switched actions twice, prompting subjects to infer their reliability. Willingness to follow informants' indications did not differ between reliable and unreliable informants. However, reduced following in later trials suggests a response to uncertainty or an undermining defeater.

Many philosophers maintain that nonverbal or minimally verbal subjects cannot be considered rational on the grounds that they lack the ability to reflect on and re‐evaluate their reasons for beliefs. This suggestion is based on the notion that reflection is the defining feature of rational thinking and is tied to relatively sophisticated linguistic skills, as we typically re‐evaluate reasons by addressing questions of the form “why does X believe that P?” (Boyle 2018; Korsgaard 2018). Making the capacity to reply to such questions a necessary condition for rationality—and thus for *reflective belief revision*—puts the threshold around the so‐called “age of reason” between 5 and 7 years old. However, this view struggles to account for human infants before they develop these linguistic abilities (Melis 2025) and altogether excludes nonhuman animals (henceforth animals) from being considered rational. By contrast, empirical researchers in developmental psychology and comparative cognition commonly accept and refer to nonlinguistic subjects as rational in a variety of characterizations (e.g., Buttelmann et al. 2008; Gergely et al. 2002; Kimura and Gopnik 2019; O'Madagain et al. 2022). There are therefore two outstanding questions: 1) Are subjects commonly thought to be unreflective, such as young children and animals, capable of rational thought? 2) If so, how does their rationality relate to the apparently more demanding capacity that some philosophers tie to language and reflection? In this paper, we build on work providing an affirmative answer to the first question, and we explore one way to answer the second.

We characterize rationality as the capacity to form beliefs in response to reasons or evidence, requiring subjects to appreciate that following the evidence is the right thing to do. Philosophers who link rationality to developed linguistic abilities maintain that such appreciation demands articulating one's reasons. However, contemporary epistemology, influenced by cognitive research with young children and animals, has become open to *unreflective* responses to evidence (Conee and Feldman 2004; Kornblith 2012; Williamson 2000). In this unreflective view of rationality, the appreciation that following the evidence is the right thing to do may remain implicit, without the ability to express it in thought or language. This unreflective characterization may suggest that young children and some animals are rational, but it does not address the relation between unreflective and reflective responses to evidence, which is crucial for assessing whether humans are unique with respect to rationality. Advocates of the more demanding notion of rationality allow that unreflective subjects respond intelligently to changing information, but argue that without the reflective capacities to think of reasons *as reasons*, and thereby to justify beliefs, they lack the self‐understanding required for rationality as it is applied to cognitively developed humans (e.g., Boyle 2018; Korsgaard 2018; Marcus 2022). Ultimately, the view that linguistically developed subjects are unique in rationality conceptualizes reflective minds as a different kind of mind, even in basic capacities (Boyle 2018; McDowell 1994). The “dualism” between unreflective and reflective minds (Bar‐On 2009) contrasts with the idea that human and animal mental faculties differ in degree rather than kind (Darwin 1872), and would be challenged by empirical evidence suggesting that reflective responses are grounded in unreflective ones, which this study tests. For a full discussion of how empirical research informs the philosophical debate on rationality, see Melis and Blakey (2025).

Here, we build on and explore the connection between unreflective and reflective belief revision. *Unreflective rational belief revision* occurs when subjects form and revise beliefs based solely on relevant evidence, without explicit reasoning (verbal or nonverbal). This manifests in at least two ways. First, it occurs in response to common positive reasons for belief (positive evidence), such as forming the belief that there are apples in the cupboard upon seeing the apples there. Second, it occurs in response to overriding evidence, such as replacing the original belief with its negation—there are no apples in the cupboard—upon going back to the cupboard and seeing *no* apples. This second case illustrates what in the epistemological literature is called an “overriding (aka rebutting) defeater”. Epistemic defeaters—such as overriding defeaters—are different to positive evidence because they provide information that contrasts a belief that is currently held. Responses to overriding defeaters can be entirely unreflective as they do not require assessment of evidence. That is, subjects can revise their beliefs automatically in response to an overriding defeater without considering what makes them do so or whether this evidence supports doing so. Later in development, humans can also reflect on their responses to overriding defeaters: they can identify their reason to give up a belief and assess this evidence as negating their current belief. Little research has addressed how this transition from unreflective to reflective belief revision occurs. We propose that a promising avenue for assessing the transition from unreflective thinking to a basic form of reflective thinking in nonverbal or minimally verbal subjects is through exploring how they respond to epistemic defeaters (Pollock 1974); specifically, “undermining (aka undercutting) defeaters” (Melis 2014).

Overriding defeaters suggest that one should replace one's belief in P with a belief in not‐P (e.g., replacing the belief there are apples in the cupboard with the belief there are no apples in the cupboard). By contrast, undermining defeaters give one reason to give up one's belief in P, or reduce one's confidence in P, without suggesting that one should believe that not‐P. Undermining defeaters may take various forms, but they often work by suggesting that something is wrong with the way in which one's belief that P has been formed, which is the type of undermining defeater we are concerned with. An undermining defeater may suggest that there was something wrong with the source of the evidence (i.e., perception, testimony, reasoning, memory), such as it being unreliable. For example, if discovering that someone had put a hallucinogenic pill into your morning coffee, this would give you a reason to doubt that your perception was reliable when you opened the cupboard and saw apples. Therefore, you acquire an undermining defeater that suggests that the source of your evidence, in this case your perception, was unreliable. In turn, the suggestion that a particular source of evidence is unreliable demands that one disregard, or be at least suspicious of, the next piece of evidence provided by this source. This means that, to respond to an undermining defeater, a subject has to (1) identify a piece of evidence as evidence (i.e., acknowledge that the evidence—seeing an apple—was the reason to form a belief), and (2) assess the evidence as likely to be misleading (e.g., evidence coming from an unreliable source—perception affected by drugs—may be misleading; Melis and Monsó 2023). As these are the distinctive features of reflective belief revision (Korsgaard 2018), responding to undermining defeaters is a basic form of reflection. Therefore, evidence that young children and animals can respond to undermining defeaters would suggest that reflection is more widespread than commonly thought and does not necessarily require language (Melis and Blakey 2025). Perhaps more importantly, studying undermining defeaters can inform us about how unreflective belief revision transitions into reflective belief revision, as undermining defeaters can be acquired through making inferences (e.g., generalization) based on overriding defeaters. This means that the reflective ability to acquire and respond to undermining defeaters is grounded in unreflective responses to overriding defeaters. Demonstrating that reflection can be based on responses to overriding defeaters coming from the same source would help us explain how the transition from unreflective to reflective belief revision happens.

To illustrate how making inferences based on overriding defeaters can lead to acquisition of an undermining defeater, imagine that you have just started a new job and are getting to know your new colleagues. Over time, you notice that a particular colleague made commitments such as attending meetings, joining for lunch, or sending documents, however, you discover that they did not always follow through on their commitments. On the basis of these experiences, you conclude that the colleague is not trustworthy. Therefore, you have *acquired* an undermining defeater like <the source is unreliable> from a series of overriding defeaters (failures to attend meetings, join for lunch, and not sending documents). Rather than continuing to believe that they will fulfill their commitments, the next time this colleague makes a promise you suspend judgment in response to the undermining defeater. Although it would be possible to ask adults why they suspend judgment, this is not an option for young children and animals. However, we could determine whether such an undermining defeater has been acquired, by examining how a subject responds to new evidence coming from the same unreliable source. The acquisition of an undermining defeater should lead to the reluctance or refusal to follow new evidence offered by the same unreliable source, without having to learn again that it is unreliable (Melis and Blakey 2025).

No studies have so far engaged directly with the question of whether nonverbal or minimally verbal subjects can acquire and respond to undermining defeaters. However, there are several studies that are relevant for engaging with the broader question of whether such subjects can identify and assess reasons. Research into social discourse and testimony has explored how children respond to information provided by others in hope of understanding how they reason about the reliability of that information, how they support their own claims with reasons, and how they evaluate reasons given by others. Commonly referred to as “selective trust”, even infants (from 8 months) demonstrate preferences for following the gaze of, asking, endorsing, or learning from reliable or accurate sources over unreliable or inaccurate sources (Chow et al. 2008; Dautriche et al. 2021; Harris et al. 2018; Rakoczy et al. 2009; Tong et al. 2020; Tummeltshammer et al. 2014). Great apes and dogs have also been found to be sensitive to the accuracy or reliability of informants and misleading evidence (Pelgrim et al. 2021; Schmid et al. 2017; Takaoka et al. 2015). However, the cues or testimony used in selective trust studies are often the same across familiarization and test phases of tasks, leaving open whether they require subjects to identify and assess reasons (e.g., by consciously evaluating the reliability of a source) or if they allow for responding to associations in the familiarization phase. Thus, it could be argued that not changing the context may allow subjects to respond based on associations or heuristic biases (Blakey et al. 2021; Melis and Blakey 2025) that do not require reflective thinking. One study that did impose a change of context was conducted by Kidd et al. (2013), first establishing the reliability of a researcher who either made true or false promises and then assessing 3‐ to 5‐year‐olds' responses in a delayed gratification task with that researcher. Results showed that children who experienced a reliable researcher waited longer than those who had experienced an unreliable one, suggesting some degree of reflection on the reliability of the researcher.

Young children have been found to revise their beliefs in response to new evidence (Gopnik and Sobel 2000; Kimura and Gopnik 2019; Kushnir and Gopnik 2007), often replacing their previous belief in favor of one suggested by the new evidence (response to overriding defeaters). More recently, researchers have started investigating the role of epistemic defeaters in explaining rational belief revision in both children and animals. For example, O'Madagain et al. (2022) aimed to investigate the ability of 3‐ to 5‐year‐old children and apes to examine one's existing beliefs and assess the justification for those beliefs against new evidence. To do this, they explored whether subjects would seek additional information (peek) when evidence (physical or social) conflicted with their initial belief about the location of a larger reward. However, though this study goes beyond previous information‐seeking paradigms testing for behavior in situations of uncertainty, it tests for overriding defeaters that challenge prior beliefs rather than undermining defeaters. Schleihauf et al. (2022) examined how 4‐ and 5‐year‐old children revise their beliefs in response to confirming and disconfirming “meta‐reasons” (reasons about reasons). The children were more likely to revise their beliefs when presented with a disconfirming meta‐reason suggesting their reason for belief was not justified (i.e., undermining defeater), and to maintain their initial beliefs in response to confirming meta‐reasons that supported their reasons for belief (i.e., positive evidence). This demonstrates that it is possible to assess undermining defeaters empirically in verbal children; however, it does not address whether undermining defeaters can be processed by nonverbal or minimally verbal subjects such as very young children or animals. Though Király et al. (2018, 2023) did not explicitly test epistemic defeaters, they did find that from 18 months, children revised attributed beliefs (beliefs about another's mental state) when confronted with evidence that undermined their initial belief attribution, offering some evidence that very young children may be capable of processing undermining defeaters.

In the current study, we aimed to develop a method to investigate whether populations that do not have—or have limited—linguistic abilities can identify and assess their reasons for belief by responding to undermining defeaters. To do this, we explored whether nonverbal or minimally verbal subjects could infer the reliability of different sources of evidence by recognizing that the evidence coming from a particular source was misleading after having been repeatedly exposed to overriding defeaters coming from that source. In this study, we used an object‐search task to expose subjects to a series of trials in which they watched a human informant (source of evidence) use an action (evidence) to indicate one of two possible reward locations before choosing where to search for it. The actions of a reliable informant reliably indicated the reward location, while the actions of an unreliable informant were independent of the reward location: they were contingent with the reward location in 50% of the trials but misleading in the other half of the trials. The task was designed to offer subjects the opportunity to make inferences about the reliability of different informants over repeated experiences with each of them. The informants used three different actions (crouching, lifting and sound) to give subjects the opportunity to understand that different forms of evidence coming from the same source can be misleading, thereby concluding that the source of the evidence is unreliable. Following this, subjects experienced four Transfer tasks that each pit the Reliable and Unreliable informants against one another to examine whether subjects could apply the acquired undermining defeater in increasingly different contexts by examining preferences for the Reliable informant.

We chose to compare 2‐year‐old children with dogs and pigs, with the expectation that all three species would demonstrate this basic form of reflective thinking. We selected 2‐year‐olds specifically because they are below the threshold of articulating reasons for their beliefs yet mobile enough for a setup comparable to animals. Dogs were chosen because they can be integrated in two‐choice experiments using human cues most comparably to human children (Guran et al. 2024; Lakatos et al. 2009). Even though it appears that dogs phylogenetic distance to humans is sufficiently distant to test ambitious claims about the link between human and animal rationality beyond primates, some may argue that due to domestication they have gone through a convergent evolution and have evolved a human‐like cognition (Hare and Tomasello 2005). However, this argument does not apply to pigs, a species bred for the purpose of meat production. Therefore, as we had access to a pig population kept and trained for behavioral research, we decided to include both species to seek broader evidence for animal rationality.

Given that both dogs and pigs follow human cues and take humans' attentional direction into account (Bensoussan et al. 2016; Catala et al. 2017; Lakatos et al. 2012; Nawroth et al. 2013, 2014), we assumed that observing an informant act on a location would support a belief like <a reward is behind the screen the informant acted on> in all three species. This belief could then be confirmed by positive evidence in all trials with the Reliable informant when subjects chose that location and found the reward behind the screen. In other trials, when the Unreliable informant's actions did not indicate the location of the reward, subjects could discover that the reward was not behind the screen the informant had acted on. A (unreflectively) rational response to these misleading trials would be to replace the belief <a reward is behind the screen the informant acted on> with its negation <a reward is *not* behind the screen the informant acted on>, thereby responding to an overriding defeater. This, in turn, predicts increasing suspicion in following the evidence (action) provided by the Unreliable informant. However, responses to overriding defeaters in a single action (one type of evidence) would not necessarily indicate acquisition of an undermining defeater. Within a particular action, increased suspicion of the evidence provided by an informant who has been unreliable in their use of that action may be an outcome of unreflective belief revision. A subject that progressively grows tired of following a particular action of an unreliable informant may just be responding to the evidence without identifying it as evidence and assessing it as likely to be misleading. An undermining defeater is acquired when an inference, based on previously acquired overriding defeaters, is made about the reliability of the informants. Hence, not until the Unreliable informant uses a different action would we have the first opportunity to see whether subjects have recognized that *any* evidence coming from that source may be misleading. We wanted to observe whether after being misled several times, subjects could identify an informant as unreliable, and on that basis, be less willing to follow their indications when they used a new action, while continuing to follow indications of a reliable informant using the same new action.

The responses we were interested in were subject's willingness to follow evidence (yes or no), the latency to make a choice, evidence following, and choice latencies in the *first trial* with each informant in each action, whether additional information was sought (via peeking) before making a choice, and whether subjects displayed a preference for the Reliable over the Unreliable informant when they were pit against one another. Our hypotheses concerning each of these measures are detailed below.

First, we were interested in the choice subjects made about whether to follow the evidence (actions of the informants). Earlier we established that young children, dogs, and to some degree, pigs show preferences for following human action and using information provided by reliable informants. Therefore, we predicted that subjects would initially follow the evidence of both the Reliable and Unreliable informants. However, after they had experienced that the Unreliable informant's actions were sometimes misleading (i.e., the reward was not in the indicated location), we expected that they would follow new actions of the Unreliable informant less often—without needing to relearn that the Unreliable informant could also be misleading in a new context—while continuing to follow the Reliable informant's evidence. We expected no species interaction with this effect, even if species had a main effect, particularly as dogs and children follow human actions more reliably than pigs. That is, we expected that all three species would infer that the informants were generally reliable or unreliable rather than treating actions independently.

Second, we were interested in how long subjects would hesitate before making a choice (choice latency) after having seen the informants' actions. We predicted that subjects would take longer to make a choice with the Unreliable informant in response to having repeatedly experienced their misleading evidence. For instance, both older children and dogs hesitate longer when faced with previously inaccurate or unreliable informants (Li et al. 2020; Takaoka et al. 2015) and we expected a similar effect in pigs as well.

Additionally, we wanted to examine subjects' responses (evidence following and choice latencies) to each informant the first time they provided new evidence (used a new action). The first time the Unreliable informant provides evidence using a new action is a pivotal moment in our paradigm. If subjects show suspicion toward the Unreliable informant while continuing to follow equivalent novel evidence provided by the Reliable informant, then this would be good evidence of a response to undermining defeaters. It would suggest that subjects had identified the evidence and assessed the evidence provided by the Reliable informant as good, and the evidence provided by the Unreliable informant as misleading, without the need to relearn that this is the case for the novel action.

We also wanted to know whether subjects were more likely to seek out additional information about the reward location in response to the Unreliable informant. Information‐seeking paradigms are often cited as evidence of metacognition (i.e., awareness of ignorance or uncertainty) in young children (Baldwin and Moses 1996; Goupil et al. 2016; Iwasaki et al. 2020), nonhuman primates (Beran and Smith 2011; Call 2010), and dogs (Belger and Bräuer 2018; Royka et al. 2020). It is unclear whether these paradigms reflect information seeking in response to subjects being in a state of uncertainty, or if they are aware of their uncertainty *as* uncertainty (Carruthers and Ritchie 2012; Perner 2012). Despite this, we included an option for subjects to seek information about the location of the reward via peeking (adopting a similar setup to Belger and Bräuer 2018) before making a choice. If subjects peeked more often in response to evidence from the unreliable informant, then this would be evidence in favor of suspicion of the Unreliable source.

Finally, we were interested in whether subjects could apply an undermining defeater in contexts that were increasingly different from the one in which it was acquired. We assessed this in four Transfer tasks (Screen Choice, Pointing, Begging, Unsolvable). The Screen Choice and Pointing tasks examined subjects' response to the informants' communicative evidence, and the Begging and Unsolvable tasks explored subjects' communication toward the informants. We predicted that subjects who had acquired an undermining defeater would prefer the Reliable informant over the Unreliable informant, following their indications and directing communication toward (e.g., requesting help) the Reliable informant.

## 1 Method

### 1.1 Subjects

We tested a total of 54 2‐year‐old children, 61 dogs, and 35 free‐ranging domestic pigs (*Sus scrofa domestica*) of the Kune Kune breed. The final child sample included 36 children aged 25 to 36 months (13 females; *M*_age_ = 30.4 months, SD = 3.6 months), recruited in central Scotland; children were predominantly British (96%) and White (98%). Data for children was collected between March 2022 and April 2023. The final dog sample included 39 dogs (18 females; *M*_age_ = 6.2 years, SD = 3 years, range = 1.2 to 12.1 years); all dogs were companion animals kept in private homes in Austria, with data collected between June and August 2022. The final pig sample included 21 pigs (9 females; *M*_age_ = 7.3 years, SD = 0.6 years, range = 6.7 to 8.9 years), with data collected between March and May 2022. Group and housing details are provided in Supporting Information.

After testing, 18 children, 22 dogs, and 14 pigs were excluded due to losing interest in the procedure (children: *n* = 6, dogs: *n* = 1), not reaching the criterion of evidence following (children: *n* = 7, dogs: *n* = 15, pigs: *n* = 14, see Procedure), researcher or technical errors (children: *n* = 3), task interference (children: *n* = 2), the testing room becoming too hot (dogs: *n* = 3), or fear‐related behaviors (dogs: *n* = 3).

### 1.2 Materials

#### 1.2.1 Experimental Setup

**FIGURE 1:**
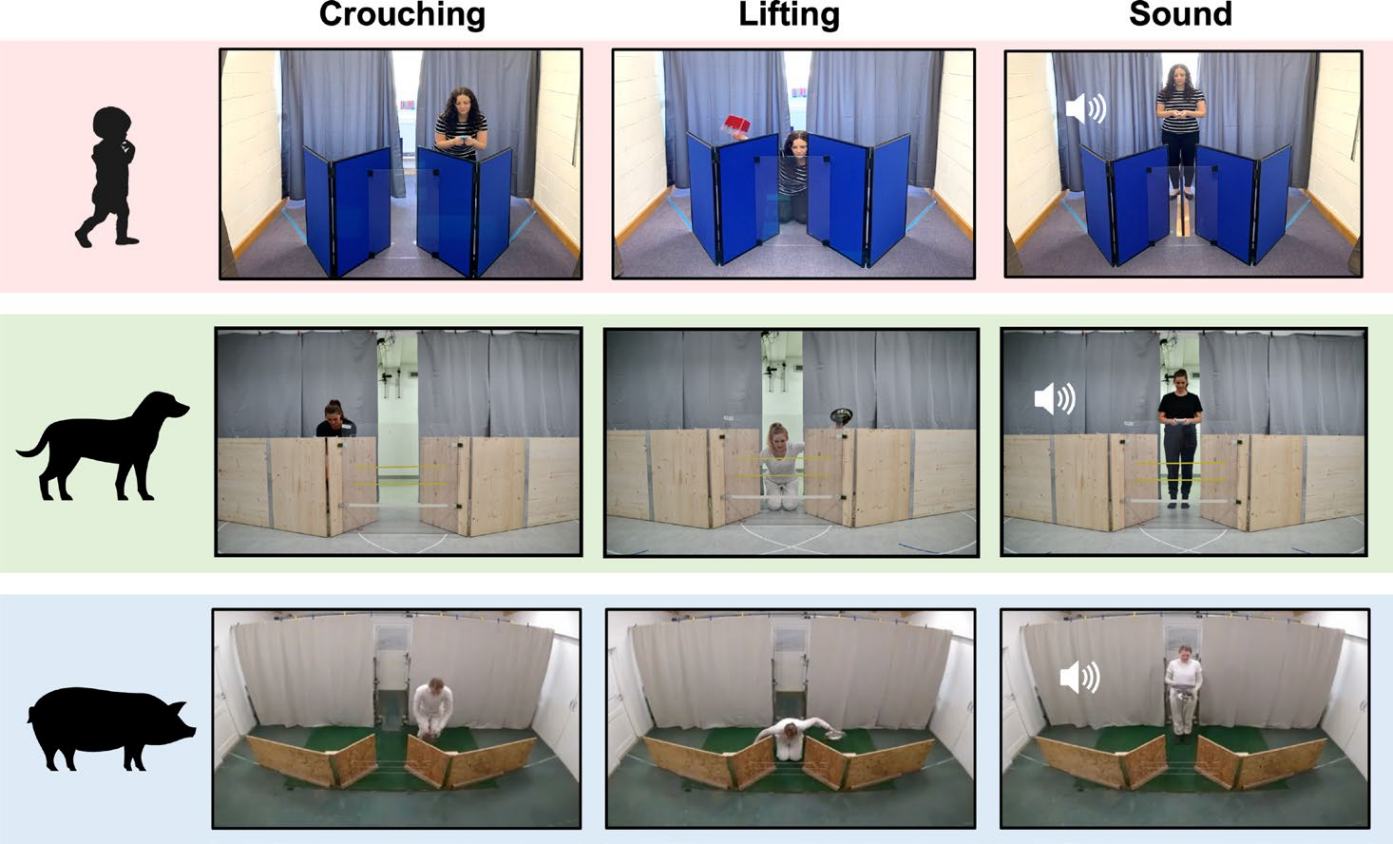
Illustration of the experimental setup for each species in each of the actions used in the Demonstration phase.

Children were tested in a room at the University of Stirling, Scotland. Dogs were also tested indoors in a room at the Clever Dog Lab, University of Veterinary Medicine Vienna. Pigs were tested at the Haidlhof Research Station, Bad Vöslau, Lower Austria, in a purpose‐built wooden hut, with an external observation area. The setup was kept as similar as possible across species. The main differences were the dimensions of the testing space and apparatus (Figures 1 and S1).

At one end of the testing room, two opaque curtains concealed the experimenters when they were not interacting with the subjects. A gap in the middle of the curtains allowed experimenters to pass through during trials. In front of the curtains, two opaque V‐shaped screens (height: 90 cm, for pigs 60 cm) with a small gap at the point of the V served as two possible hiding locations (see Figure 1). A transparent plexiglass screen or wire (pigs) was fixed between the V‐shaped screens to ensure choices were made by going around the outer edges of the screens. On the floor behind each screen, movable boards allowed experimenters to place and remove rewards from behind the curtains. To prevent subjects from making a second choice, a barrier was placed between the two screens (children: opaque board allowing visibility over the top, dogs: two wooden‐framed wire screens, pigs: permanent wire barrier). Pigs viewed actions from an observer compartment separated from the testing area by a wire mesh guillotine door, while dogs and children were held at the starting position. Rewards differed between species: children received small toys, and animals received food items (dogs: sausage or cheese, pigs: apple and bread).

#### 1.2.2 Experimenters

In each testing session, three adult experimenters played the roles of a Demonstrator, a Reliable informant, and an Unreliable informant. Each experimenter wore differently colored (white, black, striped) clothing to help subjects differentiate between them. Due to COVID‐19 regulations, all experimenters wore white FFP2 masks with children.

Children and dogs were accompanied by a caregiver. Caregivers wore a cap and looked to the ground during trials, lightly holding the subject until the signal was given to let them make a choice. Pigs were accompanied by a familiar researcher who stood outside the observer area. Additionally, children were accompanied by a fourth experimenter who was positioned on the left side of the child throughout testing and looked down during trials.

#### 1.2.3 Counterbalancing

All testing instructions (informant, action, action side, and reward side) were presented to the experimenters trial‐by‐trial on a touchscreen device running a custom program developed in PsychoPy (Peirce et al. 2019). This program randomized: the informant role of each experimenter (Demonstrator, Reliable, Unreliable) and the colors they should wear (black, white, and striped) for each subject. Informant roles and colors were consistent within subjects but varied between subjects. Other variables were pseudorandomized: the screen (left, right) behind which the action (crouching, lifting, sound) should be performed, and in the case of the Unreliable informant, whether the reward should be hidden at the indicated side. These were constrained to ensure a maximum of two consecutive trials with the same action side or reward side.

### 1.3 Procedure

The procedure consisted of three phases: the Familiarization phase; the Demonstration phase; and the Transfer phase. The task included 62 trials for dogs and pigs, and 58 trials for children (two fewer trials in the Familiarization and Transfer phases). Each subject was tested once, with all test trials completed on the same day. All dogs and half of the pigs (*n* = 10) completed an additional pre‐training prior to the Familiarization phase; the purpose of this was to afford animal subjects longer to learn to follow the first action that most children spontaneously followed. Following the evidence was defined as making the choice to search the location where the informant acted. Subjects were included in analyses if they met the evidence following criterion—following the evidence in at least three of the first four crouching trials with the Demonstrator. This criterion ensured that subjects initially followed the evidence and provided a comparable starting point across species, offsetting differences in sensitivity to human cues.

#### 1.3.1 Pre‐Training (Dogs and Pigs)

Pre‐training began with four *introductory trials* that introduced subjects to the general procedure. Subjects were led to the outer edge of the screen where the Demonstrator was standing. The Demonstrator then crouched and placed a reward, allowing the subject to retrieve it. This was repeated twice on each side (pseudorandomized order) with the subject returning to the starting position between each trial. Pre‐training continued with a series of *training trials* in which the Demonstrator used a crouching action to hide a reward behind a screen before the subject was released to search for the reward. After making a choice, the subject was called back to the starting position. All pre‐trained subjects completed at least 10 training trials, at which point their performance was measured against a predefined criterion level. To meet the criterion, the number of trials in which the subject followed the evidence (i.e., chose the side where the informant had crouched) had to be significantly greater than the number of trials in which they did not follow the evidence, based on binomial tests. After 10 trials, training continued until the criterion was reached or the subject reached the maximum number of trials, which was 20 for dogs and 40 for pigs (in two blocks of 20). Dogs took a short break after the training phase, and pigs took longer breaks of up to 1 day between each training block and between pre‐training and the subsequent phases.

#### 1.3.2 Familiarization Phase

Subjects experienced four trials (two for children) to familiarize them with the general procedure. For dogs, these were the same as the pre‐training introductory trials, while for pigs the familiar researcher rather than the Demonstrator performed the crouching action. Parents led children to the outer edge of the screen, and the fourth experimenter performed the crouching action and encouraged the child to retrieve the reward. All subjects then experienced two trials introducing them to the gap in the screen (i.e., peeking to check for a reward). In these trials, the reward was held close to the gap at the point of the screen and moved up and down until the subject that had approached from the other side followed the movement for 1 to 2 s. The reward was then placed behind the screen.

Before the Demonstration phase, the Demonstrator, Reliable informant, and Unreliable informant emerged together from behind the curtains, standing next to each other for 2 to 3 s before returning behind the curtains. The experimenters stayed in the same roles throughout all trials with a single subject.

#### 1.3.3 Demonstration Phase

The order of the 44 Demonstration phase trials was fixed, beginning with 18 crouching trials, followed by 16 lifting trials and 10 sound trials (Table 1). The three actions were chosen to have no shared component (i.e., having learned to choose a screen based on one action did not help to discriminate between screens based on a later action) and the order of actions was designed to follow a controlled progression. It began with an informant crouching behind a screen, which clearly indicated one location, and then minimized local enhancement by the informant staying between the screens and providing weaker visual discriminative cues (the lifting action) and no visual cues at all (the sound action) about a particular side in the subsequent actions.

**TABLE 1:** Outline of trial structure in the Demonstration and Transfer phases.

	Demonstrator	Reliable informant	Unreliable informant
Demonstration phase	4 × Crouching		
		4 × Crouching	
			6 × Crouching
		2 × Crouching	
	Break offered		
	2 × Crouching		
	2 × Lifting		
		4 × Lifting	
			6 × Lifting
		2 × Lifting	
	Break offered		
	2 × Lifting		
	2 × Sound		
			4 × Sound
		4 × Sound	
Transfer phase		4 × Screen Choice (dogs/pigs) or 2 × Screen choice (children)	
	Break offered		
		2 × Pointing	
		2 × begging	
	3 × Solvable	1 × Unsolvable	

The Demonstrator was the first to demonstrate each action and was not included in any of the analyses, rather we were interested in subjects' response to the Reliable and Unreliable informants. Each block of trials began with four Demonstrator trials, two repeating the previous action and two demonstrating the new action, followed by trials with the Reliable and Unreliable informants. In the crouching and lifting trials the six Reliable informant trials were split into four trials before and two trials after the six Unreliable informant trials, so that the subject concluded each block of trials with the Reliable informant. To mitigate potential order effects, the order of the informants was reversed in the sound trials (see Table 1 for trial structure). Regular breaks were offered, with additional breaks taken as needed for children.

Each trial in the Demonstration phase followed the same structure. Subjects observed an informant use an action to indicate one of the two screens. The barrier was then placed between the screens, and a knock or beep signaled that the subject could be released to make a choice. If subjects chose the rewarded side, they could eat (dogs and pigs) or retrieve the reward and put it into a container (children). If they chose the unrewarded side, experimenters removed the reward from behind the other screen by pulling back the board with the reward on it. The experimenters aimed to let the subject see the reward being removed.

The actions of the Demonstrator and Reliable informant consistently indicated the reward location, while the actions of the Unreliable informant were independent of the reward location and sometimes misleading (indicating the reward location in 50% of trials). In misleading trials, the reward was placed behind the opposite screen to the informant's action. The Unreliable informant's first trial in each action was always misleading.

##### 1.3.3.1 Crouching Trials

The informant stepped out from the curtains holding the reward in front of their body with both hands. After stepping to one side, they crouched behind the screen, placing the reward at the end of the moveable board and walked behind the edge of the curtain on the same side. In misleading trials, another informant (behind the curtain) covertly placed the reward behind the opposite screen by sliding the moveable board backwards, placing the reward, and returning the board behind the screen.

##### 1.3.3.2 Lifting Trials

The informant stepped out between the curtains holding the reward in both hands. They moved their hands behind their back (keeping the reward on the side where the action was to occur), knelt between the screens, and put an arm behind each screen. They then placed the reward on the board and lifted a container (children: red plastic tub, animals: metal food bowls) above the screen for a few seconds before setting it back down. The informant stood up, stepped backwards, and behind the curtains on the same side as they lifted the container. In misleading trials, the informant placed the reward behind the opposite screen from where they lifted the container.

##### 1.3.3.3 Sound Trials

The informant stepped out between the curtains holding a sound‐object that was not the reward but would be used to indicate the location of the reward (dogs: a plastic food container filled with dry food, pigs: a food bowl and lid, children: a white doorbell button). Using the same method as the misleading crouching trials, an informant (hidden behind the curtain) covertly placed the reward. Once in position, the informant pretended to make a sound (or for children, pressed the button). At the same time, an experimenter behind the curtains made the relevant sound using the same sound‐object (i.e., shaking the food container three times for dogs, banging the bowl and lid together three times for pigs, squeezing a squeaky toy twice for children) on the same side as the hidden reward (or the opposite side for misleading trials). The informant then stepped backward and behind the curtains to the side where the sound was made, and the sound was repeated.

#### 1.3.4 Transfer Phase

**FIGURE 2:**
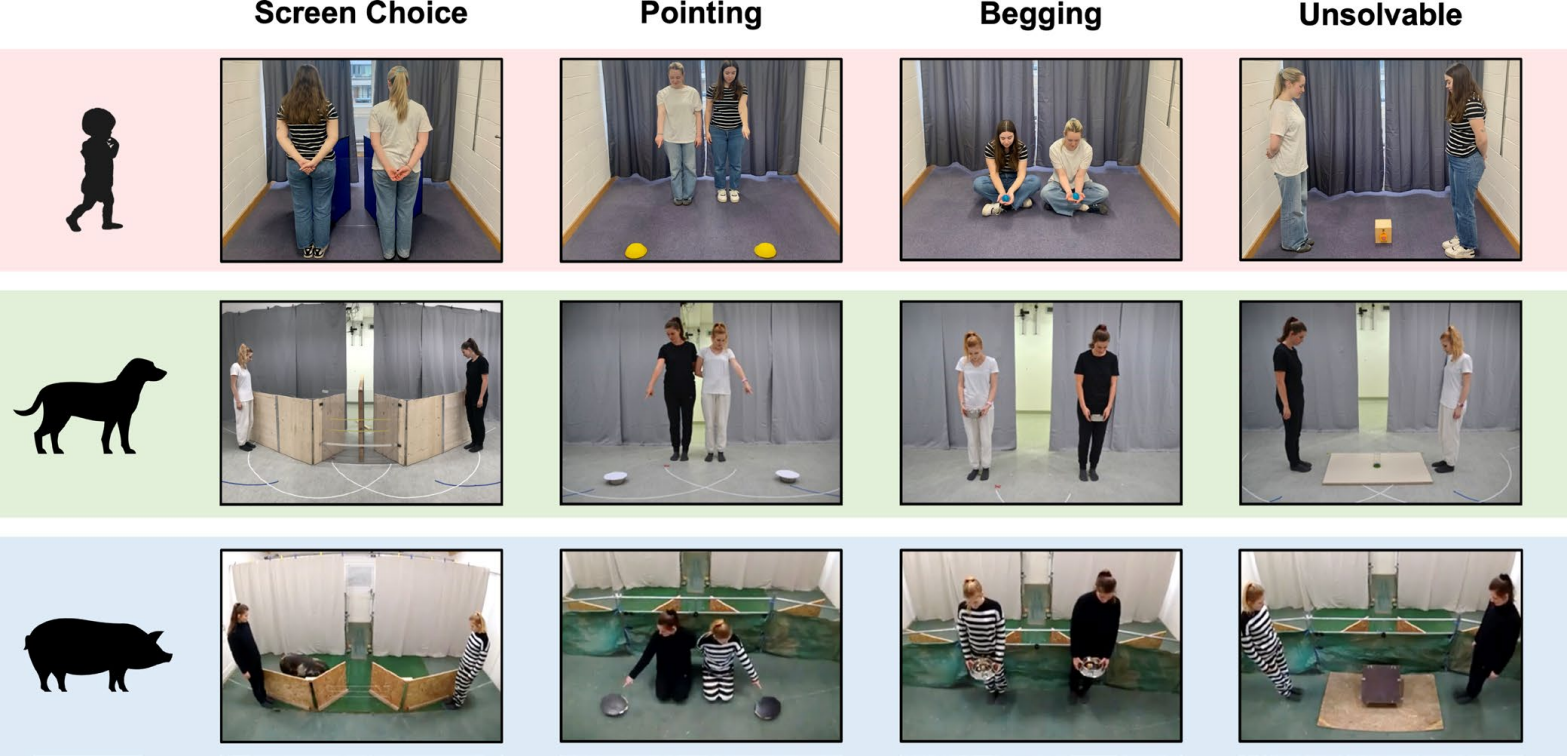
Illustration of the experimental setup for each species for each of the tasks used in the Transfer phase.

After the Demonstration phase, subjects completed four Transfer tasks: Screen Choice, Pointing, Begging, and Unsolvable (Figure 2). Each task pitted the Reliable and Unreliable informants against one another, aiming to assess any preferences for the Reliable informant. The contexts became increasingly different from the Demonstration phase to assess whether knowledge about the informants' reliability could be transferred to new contexts.

##### 1.3.4.1 Screen Choice

The Reliable and Unreliable informants stepped out from the extreme ends of the curtains and stood in front of the respective screen, looking down. Meanwhile, the Demonstrator covertly placed a reward behind one or both screens and signaled that subjects could make a choice. The location indicated by the Reliable informant was always rewarded, but the location indicated by the Unreliable informant was rewarded in 50% of trials; thus, both locations were rewarded in 50% of trials. After, the screens were removed or hidden.

##### 1.3.4.2 Pointing

The Demonstrator placed two covered bowls on the floor (spaced approx. 1 m apart for children or the width of the informant's arm span apart for dogs and pigs). The Reliable and Unreliable informants emerged from the extreme ends of the curtains, stood or knelt next to each other, and each pointed to and gazed at their closest bowl until the subject made a choice or 30 s had passed. The second bowl was immediately removed to prevent a second choice. The chosen bowl was removed when informants returned behind the curtains. The reward structure was the same as the Screen Choice task.

##### 1.3.4.3 Begging

The Reliable and Unreliable informants emerged from the extreme ends of the curtains holding identical colored balls (children) or bowls (dogs and pigs). They sat or stood next to each other and gazed at the reward for 30 s, while subjects were free to explore the room and interact with them. The informants did not reciprocate interaction with the subjects. However, to avoid upsetting children, if a reward was requested from one of the informants, the informant allowed them to take it.

##### 1.3.4.4 Unsolvable

The Demonstrator placed a container (children: wooden box with a colored padlock, dogs: upside‐down plastic jar with a lid, pigs: wooden box with metal locks on the sides) in the center of the testing area. In each Solvable trial, the Demonstrator approached the container, turned their back to the subject, baited it with a reward, and left, allowing the subject to attempt to retrieve the reward. If the subject struggled, the Demonstrator returned to help. In the Unsolvable trial, the Demonstrator sealed the container after baiting. The Reliable and Unreliable informants emerged from the extreme ends of the curtains, faced each other with the container between them, and looked at the ground. The subject could then attempt to retrieve the reward, explore the room, or interact with the informants for 2 min (30 s for children).

### 1.4 Video Coding

All testing sessions were video recorded. Children were recorded by four HiLook turret cameras (THC‐T140); the dogs by four Panasonic HD‐Camcorders HC‐V777 and one AXIS M3057‐PLVE MkII; and the pigs by two cameras, a GoPro Hero 9 inside the room and an AXIS M3065‐V for the outside observer compartment. Videos of testing sessions with children were coded for behaviors of interest using the event logging software BORIS (Friard and Gamba 2016). The videos of testing sessions with dogs and pigs were coded using the coding tool Loopy (http://loopbio.com, Loopbio Gmbh, Vienna, Austria).

In the Demonstration phase, we measured: choice, choice latencies, and peeking. In the Transfer phase, behaviors of interest varied between tasks and species, including choice (Screen Choice, Pointing), approach (Begging), and body contact (Unsolvable). As children were reluctant to approach informants, we coded choice when children pointed or approached and coded proximity and gaze direction toward each informant. The criteria can be found on the OSF (https://osf.io/qr6ne/). Interrater reliability analyses for behaviors of interest were conducted using the *irr* package (Gamer et al. 2022) in R (R Core Team 2023). Different groups of three raters coded videos of all child testing sessions, whereas videos of 12 dogs and 10 pigs were each coded by two different raters (one blind to study aims) to assess interrater reliability. Fleiss kappa (Koo and Li 2016; McHugh 2012) and intra‐class correlation coefficients (Koo and Li 2016) showed very good or excellent inter‐rater agreement for all variables (see Supporting Information). One rater (chosen at random for each subject) was included for analyses.

### 1.5 Statistical Analysis

While we had specific hypotheses, they had not been empirically tested before, making the analyses primarily exploratory. All analyses were performed using R (R Core Team 2023). Generalized linear mixed effects models (GLMMs) were fit with binomial error structure (logit link function, optimizer “bobyqa” with 200.000 iterations). To estimate subjects' probability to follow the evidence, we fit two GLMMs using *lme4* (Bates et al. 2014)—one with the full data set, and one with only the first trial data of each informant per action. Similarly, to estimate choice latencies (log‐transformed), we fit two linear mixed effects models (LMMs) using *lme4*. In all models, we included fixed effects of informant (Reliable, Unreliable), species (dogs, children, pigs) and action number (crouching −1, lifting 0, sound 1), and all possible interactions. To control for possible side biases, the action side (left, right) was included as a control fixed effect. In both LMMs, we included another control fixed effect for peeking (yes, no), as peeking would increase choice latency. Whether subjects had experienced pre‐training was highly confounded by species, so could not be included.

To estimate subjects' probability to prefer the Reliable over the Unreliable informant in the Transferphase, we fit another GLMM with preferred informant as the dependent variable (Unreliable 0, Reliable 1). We included fixed effects of species and task number (Screen Choice 0, Pointing 1, Begging 2, Unsolvable 3), and their interaction. Additionally, we included control fixed effects for the Reliable informant side (left, right) and the trial number within each task.

To account for repeated observations of the same subjects across trials, we included subject as a random intercept with random slopes for all fixed effects and kept random effects structures “maximal” where possible (Barr et al. 2013). We included by‐color‐dyad (pairing of informants' reliability and color assignment, 6 levels) as a random intercept with random slopes for all fixed effects. The following variables were dummy‐coded and centered: species, informant, peeking, action side, and Reliable informant side. Some parameters for correlations among random intercepts and slopes in the maximal model were estimated as being essentially one, indicative of not being identifiable (Matuschek et al. 2017); therefore, they were removed. We ensured comparable model fit for models with and without correlation parameters by looking at log‐likelihoods (results in Supporting Information).

To assess collinearity among predictor variables, we calculated Variance Inflation Factors (VIFs) for standard linear models, excluding random effects and interactions, using the *car* package (Fox et al. 2023). Collinearity was not an issue (VIFs ≤ 1.32). Model stabilities were evaluated by dropping individuals or levels of color dyad from the data one at a time and comparing the estimates for these models with those obtained for the full data set. For this purpose, we used a function provided by Roger Mundry (Leibniz Science Campus, Primate Cognition, Göttingen). This revealed the models to be of good stability (results in Supporting Information). To avoid issues of multiple testing, we assessed the overall effect of the predictors of interest by comparing the full model with a null model lacking these predictors (Forstmeier and Schielzeth 2011) using a likelihood ratio test (Dobson 2002). For GLMMs, fixed effects or interactions were individually tested by dropping them individually from the model and comparing simpler with more complex models using likelihood ratio tests (Barr et al. 2013). For LMMs, we tested the effect of individual fixed effects using the Satterthwaite approximation (Luke 2017) using the package *lmerTest* (Kuznetsova et al. 2017) and a model with restricted maximum likelihood. Post hoc analyses for effects of pairwise comparisons were made using the *emtrends* and *emmeans* functions (Lenth et al. 2019). To estimate confidence intervals for model estimates, we bootstrapped model estimates using the function *bootMer* in *lme4* (Bates et al. 2014). We accepted *p* values < 0.05 as statistically significant. For full model results see Supporting Information.

## 2 Results

### 2.1 Demonstration Phase

#### 2.1.1 Evidence Following

**FIGURE 3:**
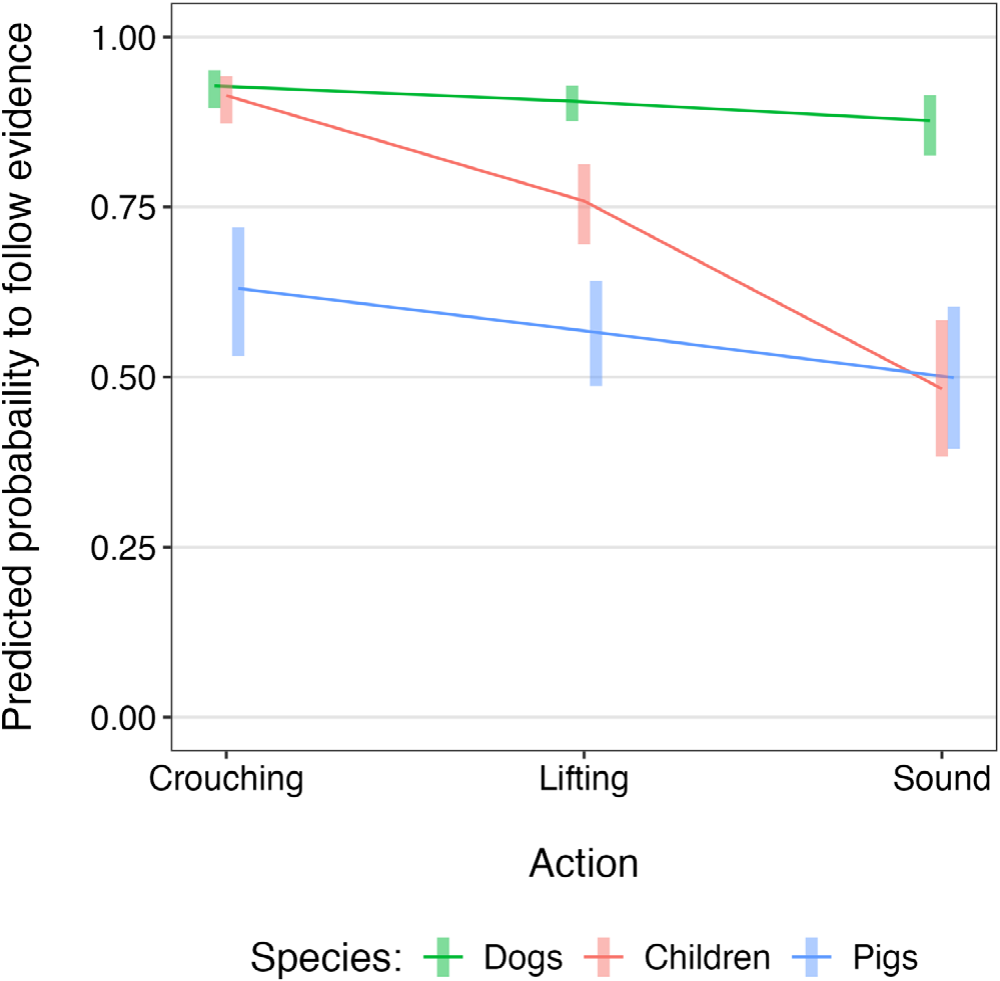
Predicted probability of each species to follow the evidence across the three actions. Confidence intervals are given for each species‐action combination at the 95% level. Results are averaged over the effects of informant and action side.

We built a GLMM to investigate whether subjects followed the evidence provided by the informants, and whether this differed between the Reliable and Unreliable informants across the different actions in the Demonstration phase. The full model was significantly better than the null equivalent that removed only the variables of interest and had the same random effect structure (*χ* ^2^(11) = 59.72, *p* < 0.001). There was no evidence of a three‐way interaction between informant, action, and species, and comparison with a reduced model without the interaction showed no significant difference (*χ* ^2^(2) = 1.33, *p* = 0.513). Examination of all two‐way interactions revealed a significant interaction between species and action (*χ* ^2^(2) = 14.77, *p* = 0.001), but none involving the informant (*p* ≥ 0.121), so these were removed. The final model showed a significant interaction between species and action (*χ* ^2^(2) = 14.64, *p* = 0.001). Post hoc analyses showed a reduction in evidence following across actions that was significant in children (*b* = −1.22, SE = 0.14, *z* = −8.74, *p* < 0.001) and dogs (*b* = −0.30, SE = 0.14, *z* = −2.13, *p* = 0.033), and marginally significant in pigs (*b* = −0.27, SE = 0.14, *z* = −1.93, *p* = 0.053). The post hoc species comparisons with regard to their change of evidence following over the course of the three actions revealed significant differences between children and dogs (*b* = 0.92, SE = 0.19, *z* = 4.90, *p* < 0.001), and children and pigs (*b* = −0.95, SE = 0.19, *z* = −4.94, *p* < 0.001), with a stronger decrease in evidence following in children compared to both dogs and pigs (Figure 3). We found no significant difference between dogs and pigs in their change of evidence following across actions (*b* = −0.03, SE = 0.19, *z* = −0.15, *p* = 0.988). However, dogs and pigs differed considerably in their average estimated probability to follow the evidence, 90.9% ± 1.3% in dogs and 57.6% ± 4.0% in pigs (mean ± SE).

#### 2.1.2 Evidence Following: First Trials

**FIGURE 4:**
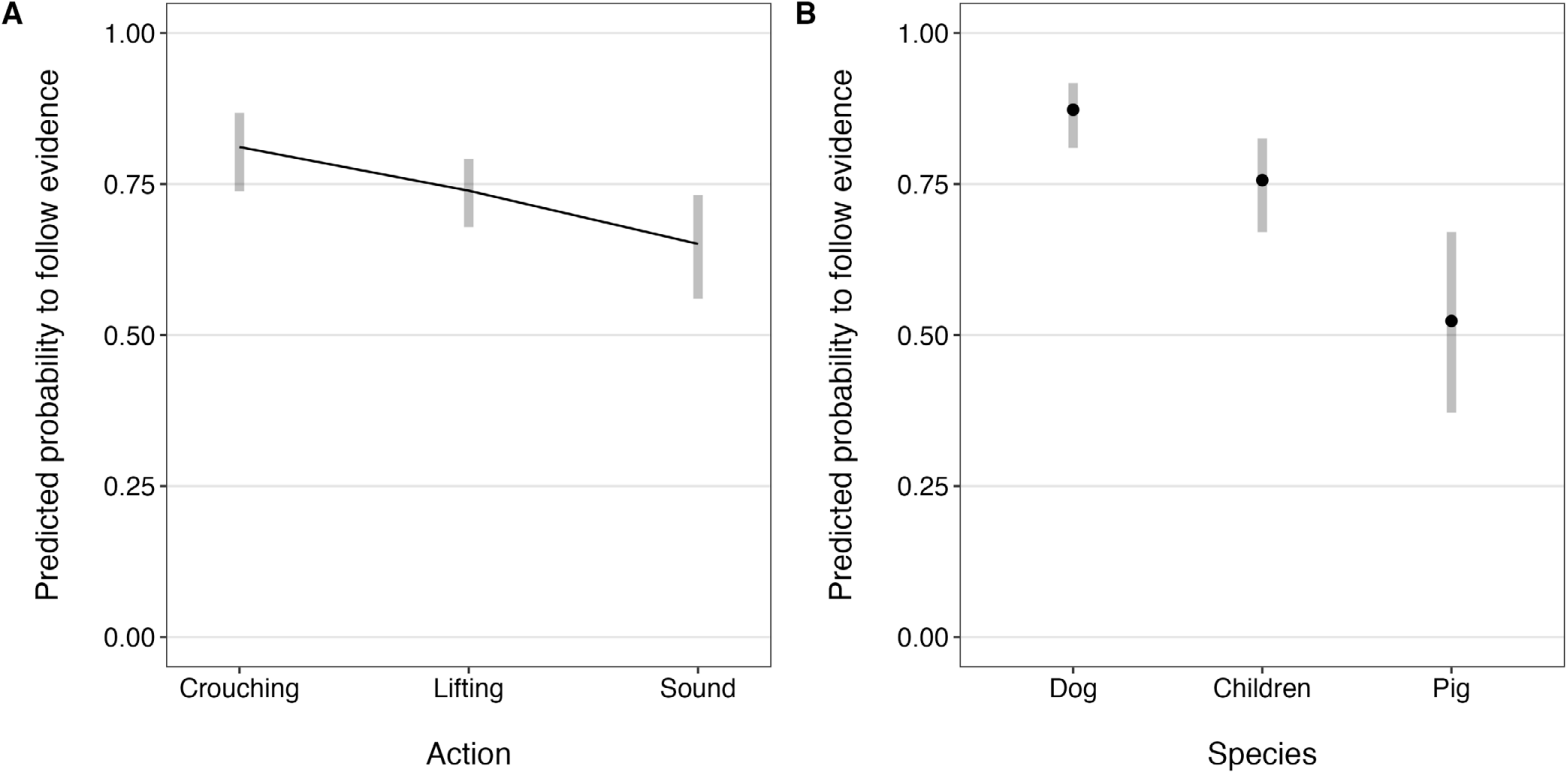
Predicted probabilities to follow the evidence in the first trials with each informant in each action. (A) Across actions (results averaged over species, informant, and action side). (B) In each species (results averaged over action number, informant, and action side). Confidence intervals are given at the 95% level.

To examine evidence following in the first trials, we built another GLMM, including only the first trial with each informant in each action. When the full model was compared to the null equivalent model, we found a significant difference (*χ* ^2^(11) = 33.45, *p* < 0.001), suggesting that the variables of interest did affect evidence following in the first trials. The model results showed no significant effects for any of the possible interactions, but there was a significant decrease of evidence following over actions (*b* = −0.44, SE = 0.14, *χ* ^2^(1) = 7.85, *p* = 0.005; Figure 4A) and a significant main effect of species (*χ* ^2^(2) = 16.67, *p* < 0.001; Figure 4B). Post hoc pairwise comparisons revealed a significant difference in evidence following between children and pigs (*b* = 1.04, SE = 0.40, *z* = 2.62, *p* = 0.024), dogs and pigs (*b* = 1.83, SE = 0.41, *z* = 4.48, *p* < 0.001), and children and dogs (*b* = 0.79, SE = 0.29, *z* = 2.70, *p* = 0.019). There were again no significant interactions or main effects involving informant.

#### 2.1.3 Choice Latencies

We were interested in whether choice latencies differed between the informants and whether this difference changed over the actions. To examine this, we constructed an LMM for choice latencies (log). There was no significant difference between the full model and its null equivalent (*χ* ^2^(11) = 19.48, *p* = 0.053), indicating that the predictor variables (informant, action and species) did not have a significant effect on the latency to make a choice.

As expected, the control predictor peeking had a significant effect (*b* = 0.80, SE = 0.10, *F*(1,4.09) = 50.88, *p* = 0.002) reflecting that it took longer to make a choice when subjects peeked. Peeking was rare and was only recorded in 111 trials with dogs (27/39), 25 trials with pigs (13/21), and one trial with children; of these, 44% were with the Unreliable informant.

#### 2.1.4 Choice Latencies: First Trials

A second LMM was built for choice latencies (log) in the first trials with each informant in each action. Here, we found a significant difference between the model and its null equivalent (*χ* ^2^(11) = 36.90, *p* < 0.001). The three‐way interaction between informant, action, and species was again not significant *F*(2,185.45) = 1.47, *p* = 0.232. Neither were any of the two‐way interactions between these predictors. We did, however, find a significant effect of species (*χ* ^2^(2) = 35.90, *p* < 0.001). Post hoc pairwise comparisons revealed significant differences between dogs and children (*b* = −0.47, SE = 0.10, *t*(6.56) = −4.50, *p* = 0.008), and dogs and pigs (*b* = −0.87, SE = 0.12, *t*(5.90) = −7.20, *p* = 0.001), but only a trend for children and pigs (*b* = −0.41, SE = 0.13, *t*(5.07) = −3.21, *p* = 0.052). Dogs were quickest to make a choice, while children and pigs took longer, regardless of the informant or action.

### 2.2 Transfer Phase

In the Transfer phase, the aim of the analysis was to examine whether subjects exhibited a preference for the Reliable informant over the Unreliable informant when they were pit against each other, and whether this preference was more pronounced in certain species and would change over the course of the Ttransfer tasks. A binomial GLMM was constructed to test for the preferred informant. The full model was not significantly different from the null model (*χ* ^2^(5) = 0.78, *p* = 0.978), indicating that the species did not differ in their preference for the Reliable informant, and that there was no overall change in choosing one or the other informant over the different Transfer tasks.

Across all subjects and tasks, the Reliable informant was chosen in 49.78% of trials. Of the 88 subjects that completed the Transfer tasks, only four subjects (1 dog, 1 pig, and 2 children) chose the Reliable informant in more than 80% of trials. Conversely, four subjects (1 dog and 3 children) chose the Unreliable informant in more than 80% of trials.

## 3 Discussion

The current study aimed to assess whether populations that do not have—or have limited—linguistic abilities can identify and assess their reasons for belief by acquiring and responding to an undermining defeater. Doing so could suggest that they have the capacity for basic reflective belief revision. Therefore, we designed a paradigm to investigate whether 2‐year‐old children, dogs, or pigs could identify and assess evidence coming from an unreliable informant as misleading and acquire an undermining defeater like <the source is unreliable> by making this inference in response to overriding defeaters. We were interested in capturing subjects' responses to the undermining defeater by refusing to follow the evidence when the same unreliable informant provided new evidence in subsequent contexts, relative to a reliable informant.

Our results indicated no difference in subjects' propensity for evidence following or their choice latency between the Reliable and Unreliable informants across the actions. There was also no evidence of a preference for either of the informants in the Transfer tasks. This was the case for all three species. Together, these results suggest that the subjects were not responding to, and had most likely not acquired, an undermining defeater such as <the source is unreliable> pertaining to the Unreliable informant. However, as outlined in the introduction, previous research indicates that young children and dogs do distinguish between informants or cues based on their accuracy or reliability. Here we suggest a few possible explanations for why our results did not show the same difference. First, unlike previous studies, the informants in our paradigm used different types of evidence in the Demonstration phase as well as further context changes in the Transfer tasks. Therefore, it could be argued that in previous studies subjects could have responded based on associations that do not require reflective thinking (Blakey et al. 2021; Melis and Blakey 2025). Second, to ensure that simply not following the evidence was not a better strategy when faced with the Unreliable informant, we made the Unreliable informant correctly indicate the reward location in 50% of the trials. Although children are more likely to avoid an informant who is wrong in many trials (Pasquini et al. 2007), 3‐year‐olds appear to lose trust in an informant even after a single error (Corriveau et al. 2009; Harris et al. 2012). Therefore, the 50% vs. 100% reliability difference should be sufficient to differentiate the informants. In a two‐choice context, subjects cannot exceed chance (50%) accuracy if they are to extract information from just observing the Unreliable informant's actions. Here, accuracy refers to the likelihood that the informant's action indicates the reward location. To put it differently, 100% inaccuracy is as informative as 100% accuracy, but the rule is reversed: subjects must choose the location not indicated. Accuracy can only exceed chance if the informant intentionally guides the choice. As such, below 50% accuracy refers to the informant's clear *intention to mislead* the subjects. In our study, the informants never addressed and did not intentionally communicate with the subjects. Thus, the Unreliable informant's 50% reliability offered the least useful evidence in a two‐choice task (i.e., minimum accuracy). Therefore, the above argument holds only if the subjects, despite not being addressed, interpreted the informants' actions as intentionally directed at them.

Even though the conditions of our study required generalization across actions, none of our three groups of subjects learned about the informants' individual reliability; we found that all three species followed the informants' actions less as the experiment progressed (with a stronger decline in children than in dogs and pigs). These results make room for the possibility that some subjects had acquired and responded to either an overriding or an undermining defeater, but that the defeater was not solely related to the Unreliable informant. Reduced evidence following across actions was already apparent in the very first trial of each action in all species. This is of pivotal interest because showing suspicion toward the Unreliable informant, but not the Reliable informant, in the very first trial of each action would be a good indication of a response to undermining defeaters. In other words, subjects would not have to relearn about the misleadingness of the evidence because they have grasped that the source of the evidence (the informant) is unreliable.

However, just like the main analysis, the first trial results did not indicate any differences in evidence following or choice latency between the informants. Instead, we observed a general reduction in evidence following in response to both informants. One explanation for this is that subjects did acquire an undermining defeater by making an inference over responses to previous overriding defeaters, but that the undermining defeater was not restricted to the Unreliable informant as the unreliable source of evidence. Although we designed the paradigm such that the informants were the sources of evidence and the action itself was the evidence, from the results it is, however, not possible to know what subjects took to be the source of the evidence. That subjects did not respond differently to the two informants suggests that they may have taken the informants together as the source of evidence. For example, subjects could still have generalized to <the source is unreliable>, but <the source> refers to both informants in the task, rather than just the Unreliable informant. Though this is not what we had intended subjects to consider, reduced evidence following with both informants across the actions could be explained by a response to an undermining defeater such as <the source is unreliable>. If subjects had generalized to such an undermining defeater, we would expect them to stop relying on the informants' indications, meaning that following the evidence would no longer be different from chance. Indeed, this is what we found in both children and pigs.

Based on these results, however, we do not have firm evidence to argue that subjects responded reflectively. The gradual decrease in evidence following may also point to the subjects' increasing uncertainty of which strategy they shall follow, possibly evoked by responding to the overriding defeaters in an unreflective manner or by the unclear contingencies of this complex experiment. Uncertainty can guide decision‐making and learning in both nonverbal human infants and animals; it is usually thought of as the quality of one's belief state encoded by unreflective or even subpersonal (e.g., purely neural) processes (Baer and Kidd 2022). Some might argue that the reduction in following the evidence across actions is the result of later actions being less visually salient indicators of the reward location than earlier actions. However, we believe our results do not support this explanation. For instance, though dogs showed a significant reduction in following the evidence across actions, they still followed the evidence in over 75% of sound trials, suggesting that the sound cue was salient to them. Additionally, we observed the strongest reduction in 2‐year‐olds, who we would expect to outperform animals in following nonverbal cues based on previous research (Lakatos et al. 2009; Moore et al. 2015). Relative to children, we observed a weaker reduction in evidence following across actions in animals. Dogs are renowned for their motivation to interact with humans, follow their initiations, and adjust to their movements (Duranton and Gaunet 2018; Feuerbacher et al. 2023; Range et al. 2019), which may explain why they followed the informants' actions more than both pigs and children. Specifically, receiving no reward in some trials might have had less impact on the dogs' behavior compared to the other species. This could account for the differences between dogs and pigs in terms of evidence following and choice latency, though differences in latency could also be an artifact of the different speeds at which dogs and pigs move.

Since our results are compatible with both reflective and unreflective interpretations, they do not resolve whether nonverbal and minimally verbal subjects are rational in the same sense as developed linguistic subjects. If subjects did acquire an undermining defeater, it would suggest that unreflective subjects can ascend to the reflective level based on their unreflective responses to the evidence, thus supporting the continuity view (difference in degree rather than kind) of rationality and challenging the claim that only developed linguistic subjects are capable of reflective rationality. However, if subjects' reduction in evidence following is better explained by unreflective responses, the difference in kinds of minds claim remains unchallenged.

If subjects are expected to ascend to the reflective level based on what they do unreflectively, presumably a lower‐level unreflective explanation of what they do will always, in principle, be available. This challenge is not unique to our study but reflects a broader issue in interpreting theoretical claims (see Povinelli and Vonk 2003). Rather than relying entirely on the results of empirical studies, understanding the relation between human and animal rationality requires consideration of different aspects of the similarities and differences between human and animal minds, including studies from neuroscience, experiments in comparative psychology, philosophical arguments about the nature of rationality, and methodological considerations. Experimental results remain crucial and, given the complexity of the issue, many of them will need to be considered before determining whether animals and humans are rational in the same sense.

This paradigm was a first step in developing a method to investigate whether nonverbal or minimally verbal subjects have the capacity for basic reflective belief revision. Therefore, we will outline some limitations that could be addressed in future studies. First, a substantial number of subjects had to be excluded from the analyses due to not reaching our criterion of evidence following in the first four trials. Our choice to exclude these subjects came from our expectation that subjects should initially follow positive evidence by choosing to search the indicated location, and that those that do not follow positive evidence would be unlikely to later respond to defeaters (overriding and undermining). Despite including pre‐training that gave dogs and pigs longer to understand the purpose of the evidence, some subjects still failed to reach this criterion. This may be because the sources of evidence were human. Although children, dogs, and pigs do all follow human evidence, having a human informant as a source may have made it more challenging for the animals to distinguish the evidence and source of evidence. Furthermore, this concern can be extended into a general criticism of the paradigm: if the subjects consider the informants themselves as part of the evidence, one can argue that even if the subjects had followed the Reliable informant more than the Unreliable one, this differentiation could be seen as a simple associative response as following the Reliable informant yields more reward and is therefore better reinforced than following the Unreliable informant.

Supporting further paradigm development, it is also important to note that our experiment offered eight misleading trials (overriding defeaters), and six of these could be used for generalization (three in crouching, three in lifting). We expected that this would be sufficient to acquire an undermining defeater if subjects had the capacity to do so; however, the limited exposure to overriding defeaters may have been too much of a challenge for our subjects, and should be addressed in future studies. Ideally, we would have included more trials, but practical limitations including retaining subjects' attention for the whole task prevented this. One way to investigate whether undermining defeaters can be acquired with limited exposure to overriding defeaters in our paradigm would be to examine human adults whom we know have the capacity to respond to undermining defeaters. Testing older children would also allow for investigation into the developmental trajectory of processing undermining defeaters and whether this is associated with linguistic ability.

Overall, this study is the first to address the possibility of assessing capacities for basic forms of reflective belief revision in nonverbal or minimally verbal populations by examining responses to undermining defeaters. The results showed differences between species regarding their evidence following across actions, which may reflect differences in motivation, speed of acquiring an undermining defeater, or of developing uncertainty. The overall reduction in evidence following we observed may suggest responding to either uncertainty or an undermining defeater like <the source is unreliable>. However, as the subjects did not distinguish between the Reliable and Unreliable informants, their behavior instead points to them having taken informants together to be the source of evidence. Whether nonverbal subjects are capable of basic reflective belief revision remains an open question to be investigated further.

## Ethics Statement

This study was approved by the General University Ethics Panel and the Animal Welfare Ethical Review Board at the University of Stirling (AWERB 2021 3029 3060) as well as the Ethics and Animal Welfare Committee of the University of Veterinary Medicine, Vienna (ETK—050/03/2022). Research was conducted in accordance with relevant guidelines and regulations, and informed written consent was obtained from children's parents and dog caregivers prior to participation.

## Conflicts of Interest

The authors declare no conflicts of interest.

## Supporting information


**Data S1:** cdev70020‐sup‐0001‐supinfo.docx.

## Data Availability

The data and code necessary to reproduce the analyses presented here are publicly accessible, as are the materials necessary to attempt to replicate the findings. The data, code, and materials for this research are available at the following URL: [https://osf.io/qr6ne/]. The analyses presented here were not preregistered.
