# Supplementary material for: Nonverbal Rationality? 2‐Year‐Old Children, Dogs, and Pigs Show Unselective Responses to Unreliability but to Different Degrees
Source: Child Dev. 2025 Aug 4;96(6):2047–61. doi: 10.1111/cdev.70020 (PMC12598444; doi:10.1111/cdev.70020)
Supplement: Supplementary file 1 — Data S1: cdev70020‐sup‐0001‐supinfo.docx. [file CDEV-96-2047-s001.docx]

# Supplementary Method

## Subjects

All pigs lived under semi-natural conditions in one life-long kin-based group consisting of 19 boars and 18 sows, and in close and constant contact to human caregivers since birth. The pigs were kept on an 8-ha pasture with a 1-ha forest at the Haidlhof Research Station, Bad Vöslau, Lower Austria. Within the forest, six insulated wooden A-shaped huts offered shelter and a wallow for the possibility for skin care and cooling down. Food was provided daily in form of vegetables and boiled corn; pigs were also free to graze on the clover-grass mixture on the pasture. Well-water was provided at two stations.

## Materials


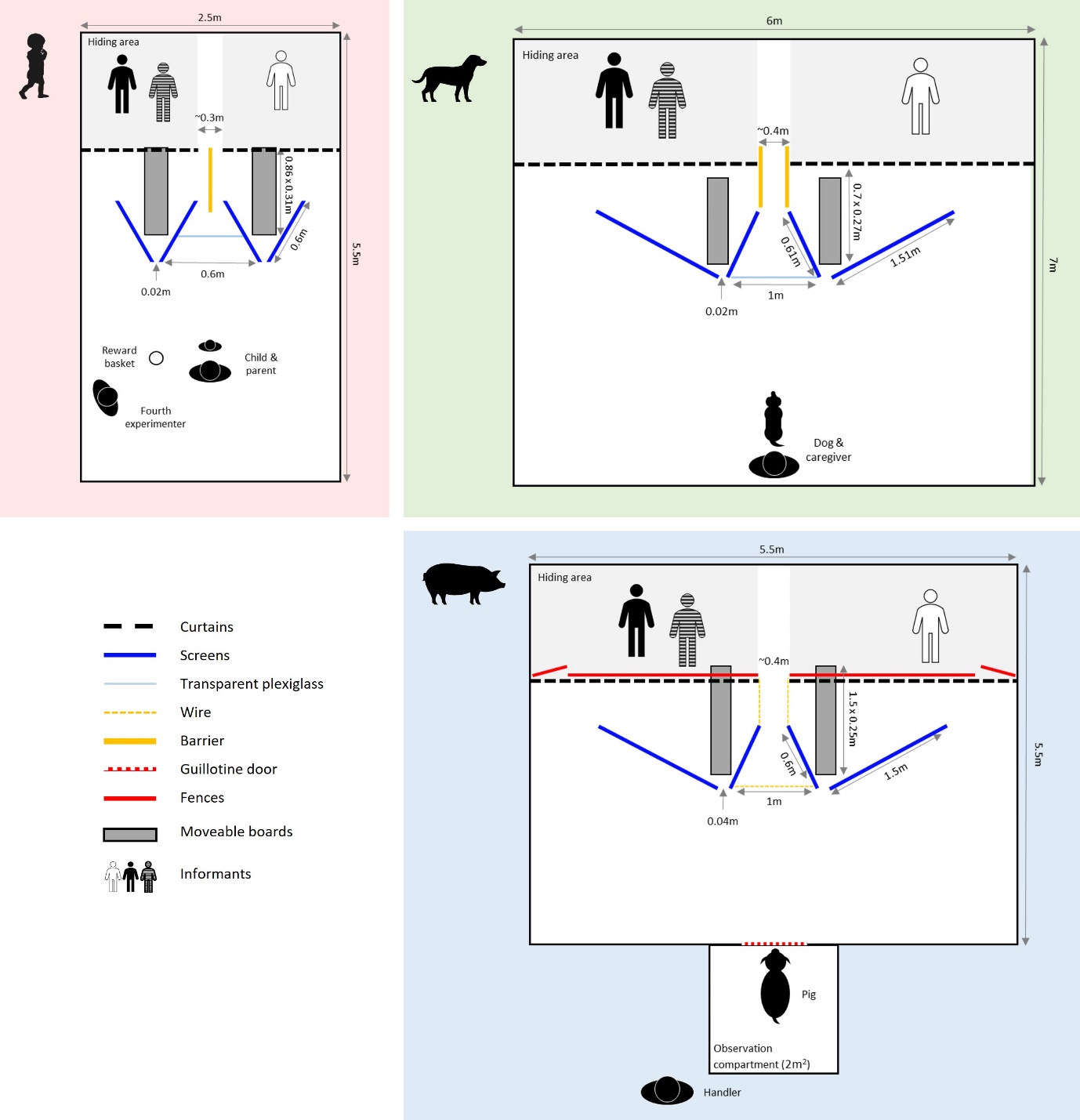
**Figure S1**Schematic representation of the experimental set-up for each species (not to scale).

**Table S1**
Demographic information for dogs included in analysis including subject ID, breed, breed type, age, and sex.

| **Subject ID** | **Breed** | **Breed Type** | **Age (years)** | **Sex** |
| --- | --- | --- | --- | --- |
| dog1 | Australian Shepherd | Cooperative | 8.7 | F |
| dog4 | Irish glen of Imaal Terrier | Independent | 7.2 | M |
| dog5 | Border Terrier | Independent | 3.3 | F |
| dog8 | Shetland Sheep Dog | Cooperative | 2.6 | M |
| dog9 | Shetland Sheep Dog | Cooperative | 11.1 | F |
| dog11 | Australian Shepherd | Cooperative | 4.3 | M |
| dog12 | Border Collie | Cooperative | 5.8 | M |
| dog13 | Australian Shepherd | Cooperative | 3.1 | F |
| dog14 | Border Collie | Cooperative | 6.8 | M |
| dog15 | Shetland Sheep Dog | Cooperative | 12.1 | M |
| dog17 | Shetland Sheep Dog | Cooperative | 4.3 | M |
| dog18 | Border Collie | Cooperative | 11.4 | M |
| dog19 | Parson Russell Terrier | Independent | 11 | F |
| dog22 | Collie | Cooperative | 6.3 | F |
| dog26 | Parson Russell Terrier | Independent | 5 | M |
| dog27 | Irish glen of Imaal Terrier | Independent | 5.6 | F |
| dog28 | Border Collie | Cooperative | 7.2 | M |
| dog30 | Border Collie | Cooperative | 10.8 | M |
| dog31 | Irish glen of Imaal Terrier | Independent | 9.3 | F |
| dog32 | Parson Russell Terrier | Independent | 2.5 | M |
| dog34 | Parson Russell Terrier | Independent | 8.4 | F |
| dog36 | Irish glen of Imaal Terrier | Independent | 2.6 | F |
| dog37 | Shetland Sheep Dog | Cooperative | 3 | F |
| dog38 | West Highland Terrier | Independent | 2.7 | F |
| dog42 | Australian Shepherd | Cooperative | 9.3 | F |
| dog43 | Parson Russell Terrier | Independent | 8 | F |
| dog44 | Border Terrier | Independent | 4.9 | M |
| dog45 | Australian Shepherd | Cooperative | 5.4 | F |
| dog47 | Australian Shepherd | Cooperative | 5.2 | M |
| dog50 | Border Collie | Cooperative | 8.8 | M |
| dog51 | Border Collie | Cooperative | 9.2 | M |
| dog52 | Australian Shepherd | Cooperative | 3.3 | M |
| dog53 | Fox Terrier | Independent | 1.3 | M |
| dog54 | Parson Russell Terrier | Independent | 1.2 | M |
| dog55 | Border Terrier | Independent | 6.6 | F |
| dog57 | Parson Russell Terrier | Independent | 4.3 | F |
| dog59 | Border Terrier | Independent | 5.9 | F |
| dog60 | Border Terrier | Independent | 8.3 | M |
| dog61 | Border Collie | Cooperative | 3.2 | M |

**Table S2**
Demographic information for pigs included in analysis including subject ID, age, and sex.

| **Subject ID** | **Name** | **Age (years)** | **Sex** |
| --- | --- | --- | --- |
| pig1 | Rasputin | 7.5 | M |
| pig2 | Beauty | 8.9 | F |
| pig4 | Zafran | 6.7 | M |
| pig5 | Barbarossa | 6.7 | M |
| pig7 | Romeo | 7.5 | M |
| pig10 | Raya | 6.7 | F |
| pig11 | Zeppelin | 6.7 | M |
| pig14 | Zoe | 7.6 | F |
| pig16 | Rosine | 6.7 | F |
| pig17 | Zaccharias | 7.7 | M |
| pig18 | Bella | 7.6 | F |
| pig19 | Zardoz | 6.9 | M |
| pig20 | Rapunzel | 7.7 | F |
| pig23 | Ronon | 6.9 | M |
| pig26 | Zafira | 7.7 | F |
| pig27 | Bruno | 6.9 | M |
| pig28 | Bibi | 7.7 | F |
| pig29 | Zeus | 6.9 | M |
| pig31 | Zampano | 7.7 | M |
| pig33 | Rudi | 7.8 | M |
| pig34 | Blume | 7.7 | F |

## Video Coding

Intra-class correlation coefficients (Koo & Li, 2016) were used to assess rater agreement for choice latencies indicating excellent agreement (ICC: children = .983, dogs = .997, pigs = .999). Ratings of choice – assessed using Fleiss kappa (Koo & Li, 2016; McHugh, 2012) – demonstrated very good agreement (kappa: children = 1, dogs = .983, pigs = .989). Although there were too few data points to assess peeking in children, rater agreement was excellent in dogs (kappa = .902) and very good in pigs (kappa = .866). The approach measure in the Begging task showed very good agreement in dogs (kappa = 1) and moderate agreement in pigs (kappa = .597), and there was excellent agreement for body contact (separate for the duration of contact with the informant standing on the left and right sides) in the Unsolvable task (ICC: dogs = .998 (left) and .999 (right), pigs = .959 (left) and .965 (right)).

# Supplementary Results

## Demonstration Phase

### Evidence Following

**Table S3A**
Results of the full GLMM investigating the probability to follow the evidence (estimates, together with standard errors, confidence limits, significance tests, as well as minimum and maximum of estimates obtained after dropping individuals one at a time).

|  | **Estimate** | **SE** | **lwr CI** | **upr CI** | **Chisq** | **Df** | **P** | **min** | **max** |
| --- | --- | --- | --- | --- | --- | --- | --- | --- | --- |
| Full null model comparison |  | | | | 59.717 | 11 | < 0.001 |  | |
| (Intercept) | 2.432 | 0.216 | 2.037 | 2.889 | ^(1)^ | ^(1)^ | ^(1)^ | 2.224 | 2.588 |
| Informant _Unreliable_ | -0.115 | 0.198 | -0.473 | 0.242 | ^(1)^ | ^(1)^ | ^(1)^ | -0.242 | -0.012 |
| Species _Children_ | -1.332 | 0.261 | -1.834 | -0.878 | ^(1)^ | ^(1)^ | ^(1)^ | -1.565 | -1.127 |
| Species _Pig_ | -2.003 | 0.231 | -2.478 | -1.560 |  |  |  | -2.238 | -1.814 |
| Action | -0.297 | 0.202 | -0.700 | 0.124 | ^(1)^ | ^(1)^ | ^(1)^ | -0.498 | -0.187 |
| Action Side _Right_ | -0.227 | 0.262 | -0.738 | 0.300 | 0.752 | 1 | 0.386 | -0.539 | -0.111 |
| Informant _Unreliable_ * Species _Children_ | 0.434 | 0.258 | -0.045 | 0.909 | ^(1)^ | ^(1)^ | ^(1)^ | 0.279 | 0.578 |
| Informant _Unreliable_ * Species _Pig_ | 0.014 | 0.270 | -0.514 | 0.522 |  |  |  | -0.140 | 0.111 |
| Informant _Unreliable_ * Action | 0.001 | 0.259 | -0.531 | 0.513 | ^(1)^ | ^(1)^ | ^(1)^ | -0.139 | 0.193 |
| Species _Children_ * Action | -0.867 | 0.271 | -1.421 | -0.380 | ^(1)^ | ^(1)^ | ^(1)^ | -1.072 | -0.766 |
| Species _Pig_ * Action | -0.118 | 0.286 | -0.677 | 0.448 |  |  |  | -0.275 | 0.196 |
| Informant _Unreliable_ * Species _Children_ * Action | -0.103 | 0.345 | -0.785 | 0.585 | 1.335 | 2 | 0.513 | -0.212 | 0.035 |
| Informant _Unreliable_ * Species _Pig_ * Action | 0.293 | 0.360 | -0.402 | 1.008 |  |  |  | -0.003 | 0.512 |

(1) not shown due to very limited interpretability

**Figure S2**


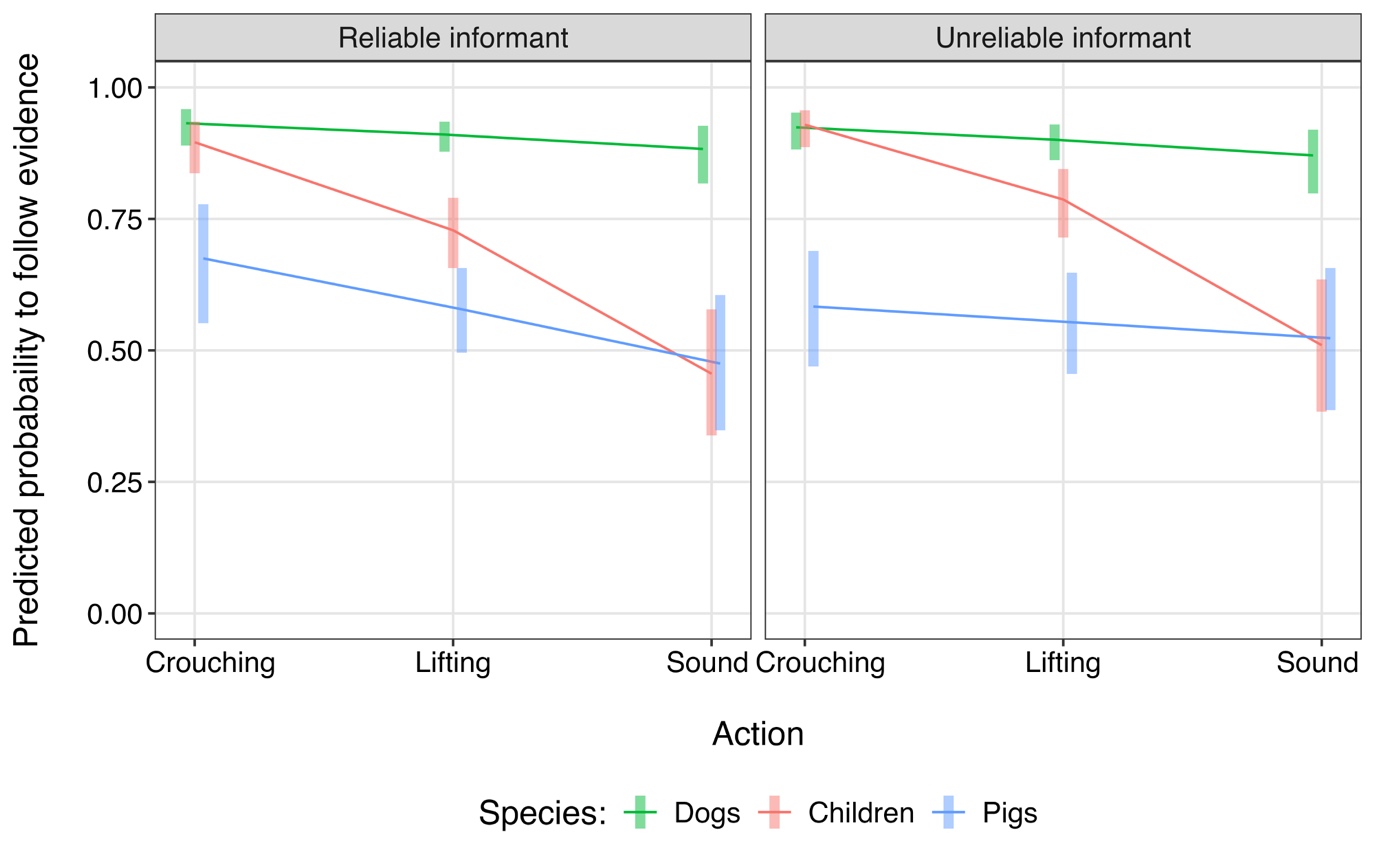
Predicted probability of each species to follow the evidence of the Reliable and Unreliable informants over the different actions.

*Note.* Confidence level used: 0.95. Results are averaged over levels of action side. The depicted three-way interaction is not significant.

**Table S3B**Results of the reduced GLMM investigating the probability to follow the evidence (estimates, together with standard errors, confidence limits, significance tests). Model reduced by removing the 3-way interaction of informant role, species and action.

|  | **Estimate** | **SE** | **lwr CI** | **upr CI** | **Chisq** | **Df** | **P** |
| --- | --- | --- | --- | --- | --- | --- | --- |
| (Intercept) | 2.436 | 0.217 | 2.074 | 2.874 | ^(1)^ | ^(1)^ | ^(1)^ |
| Informant _Unreliable_ | -0.114 | 0.198 | -0.494 | 0.294 | ^(1)^ | ^(1)^ | ^(1)^ |
| Species _Children_ | -1.329 | 0.262 | -1.843 | -0.844 | ^(1)^ | ^(1)^ | ^(1)^ |
| Species _Pig_ | -2.013 | 0.233 | -2.500 | -1.572 |  |  |  |
| Action | -0.327 | 0.163 | -0.629 | -0.029 | ^(1)^ | ^(1)^ | ^(1)^ |
| Action Side _Right_ | -0.229 | 0.262 | -0.760 | 0.264 | 0.762 | 1 | 0.383 |
| Informant _Unreliable_ * Species _Children_ | 0.441 | 0.257 | -0.099 | 0.960 | 4.218 | 2 | 0.121 |
| Informant _Unreliable_ * Species _Pig_ | -0.022 | 0.268 | -0.540 | 0.518 |  |  |  |
| Informant _Unreliable_ * Action | 0.056 | 0.149 | -0.217 | 0.358 | 0.142 | 1 | 0.706 |
| Species _Children_ * Action | -0.926 | 0.186 | -1.304 | -0.588 | 14.774 | 2 | 0.001 |
| Species _Pig_ * Action | 0.056 | 0.192 | -0.329 | 0.434 |  |  |  |

^(1)^ not shown due to very limited interpretability

**Table S3C**
Results of the final reduced GLMM investigating the probability to follow the evidence (estimates, together with standard errors, confidence limits, significance tests). Model reduced by removing any interaction involving informant role.

|  | **Estimate** | **SE** | **lwr CI** | **upr CI** | **Chisq** | **Df** | **P** |
| --- | --- | --- | --- | --- | --- | --- | --- |
| (Intercept) | 2.365 | 0.201 | 1.978 | 2.792 | ^(1)^ | ^(1)^ | ^(1)^ |
| Informant _Unreliable_ | -1.151 | 0.241 | -0.178 | 0.256 | 0.177 | 1 | 0.674 |
| Species _Children_ | -2.003 | 0.207 | -1.617 | -0.711 | ^(1)^ | ^(1)^ | ^(1)^ |
| Species _Pig_ | -0.296 | 0.138 | -2.441 | -1.631 |  |  |  |
| Action | 0.045 | 0.108 | -0.563 | -0.038 | ^(1)^ | ^(1)^ | ^(1)^ |
| Action Side _Right_ | -0.239 | 0.260 | -0.744 | 0.303 | 0.928 | 1 | 0.335 |
| Species _Children_ * Action | -0.913 | 0.187 | -1.284 | -0.57 | 14.642 | 2 | 0.001 |
| Species _Pig_ * Action | 0.051 | 0.192 | -0.312 | 0.431 |  |  |  |

^(1)^ not shown due to very limited interpretability

### Evidence Following: First Trials

**Table S4A**
Results of the full GLMM investigating the probability to follow the evidence in the first trials of each informant per action (estimates, together with standard errors, confidence limits, significance tests, as well as minimum and maximum of estimates obtained after dropping individuals one at a time).

|  | **Estimate** | **SE** | **lwr CI** | **upr CI** | **Chisq** | **Df** | **P** | **min** | **max** |
| --- | --- | --- | --- | --- | --- | --- | --- | --- | --- |
| Full null model comparison |  | | | | 33.445 | 11 | 0.0004 |  | |
| (Intercept) | 1.863 | 0.341 | 1.239 | 2.821 | ^(1)^ | ^(1)^ | ^(1)^ | 1.807 | 2.032 |
| Informant _Unreliable_ | 0.116 | 0.417 | -0.688 | 1.091 | ^(1)^ | ^(1)^ | ^(1)^ | -0.120 | 0.376 |
| Species _Children_ | -0.963 | 0.433 | -1.974 | -0.053 | ^(1)^ | ^(1)^ | ^(1)^ | -1.159 | -0.689 |
| Species _Pig_ | -2.017 | 0.525 | -3.392 | -1.112 |  |  |  | -2.168 | -1.734 |
| Action | -0.120 | 0.346 | -0.878 | 0.627 | ^(1)^ | ^(1)^ | ^(1)^ | -0.385 | 0.150 |
| Action Side _Right_ | 0.009 | 0.248 | -0.501 | 0.575 | 0.001 | 1 | 0.971 | -0.219 | 0.185 |
| Informant _Unreliable_ * Species _Children_ | 0.299 | 0.672 | -1.206 | 1.589 | ^(1)^ | ^(1)^ | ^(1)^ | -0.087 | 0.536 |
| Informant _Unreliable_ * Species _Pig_ | 0.353 | 0.645 | -0.849 | 1.709 |  |  |  | -0.077 | 0.691 |
| Informant _Unreliable_ * Action | 0.103 | 0.516 | -1.000 | 1.187 | ^(1)^ | ^(1)^ | ^(1)^ | -0.197 | 0.362 |
| Species _Children_ * Action | -0.508 | 0.449 | -1.632 | 0.401 | ^(1)^ | ^(1)^ | ^(1)^ | -0.936 | -0.175 |
| Species _Pig_ * Action | -0.607 | 0.568 | -1.977 | 0.537 |  |  |  | -1.250 | -0.257 |
| Informant _Unreliable_ * Species _Children_ * Action | -0.514 | 0.660 | -1.987 | 0.901 | 1.558 | 2 | 0.459 | -0.946 | -0.257 |
| Informant _Unreliable_ * Species _Pig_ * Action | 0.646 | 0.901 | -1.163 | 2.797 |  |  |  | 0.255 | 1.549 |

^(1)^ not shown due to very limited interpretability

**Figure S3**

Predicted probability of each species to follow the evidence in the first trials of the Reliable and Unreliable informants over the different actions.

**
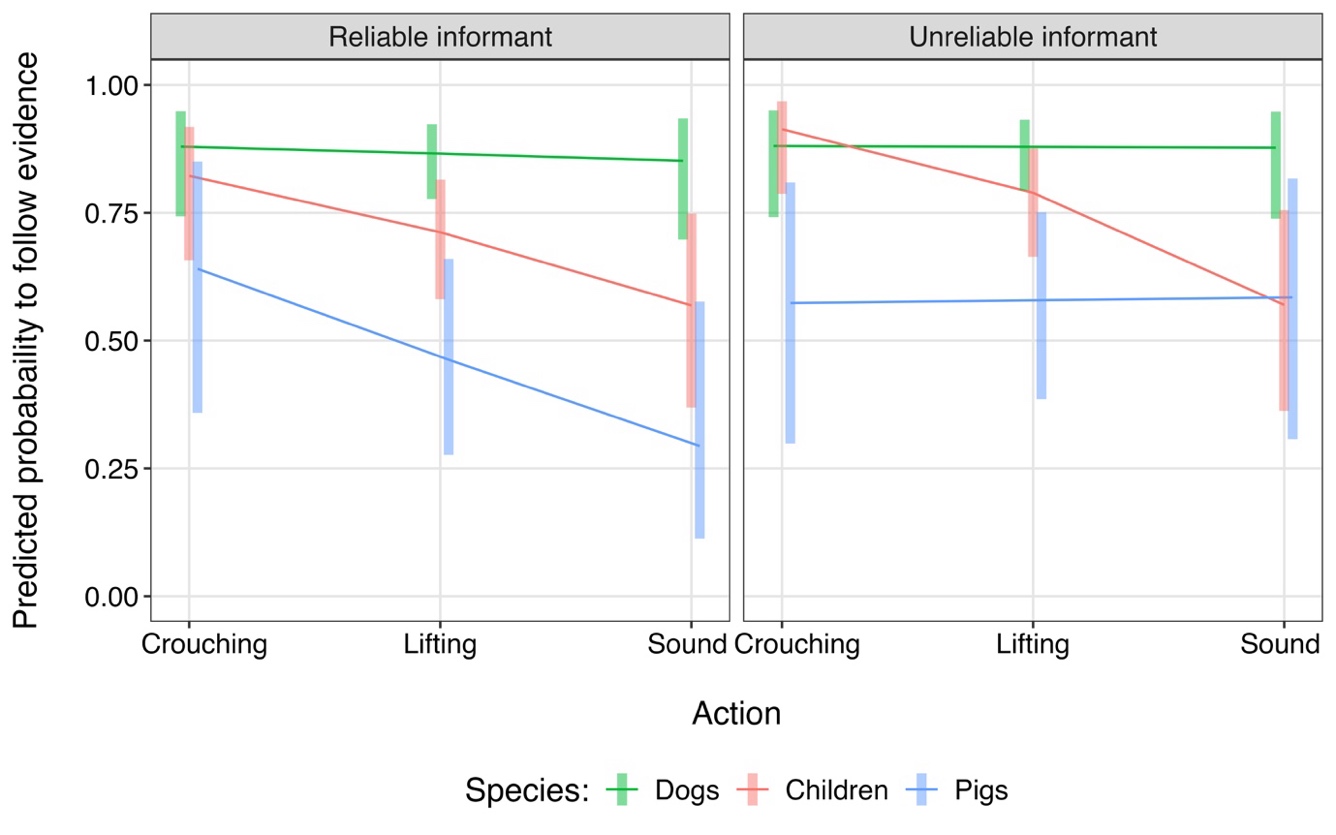
**

*Note.* Confidence level used: 0.95. Results are averaged over levels of action side. The depicted three-way interaction is not significant.

**Table S4B**
Results of the reduced GLMM investigating the probability to follow the evidence in all first trials of each informant per action (estimates, together with standard errors, confidence limits, significance tests). Model reduced by removing the 3-way interaction of informant role, species and action.

|  | **Estimate** | **SE** | **lwr CI** | **upr CI** | **Chisq** | **Df** | **P** |
| --- | --- | --- | --- | --- | --- | --- | --- |
| (Intercept) | 1.880 | 0.342 | 1.306 | 2.859 | ^(1)^ | ^(1)^ | ^(1)^ |
| Informant _Unreliable_ | 0.119 | 0.418 | -0.752 | 1.056 | ^(1)^ | ^(1)^ | ^(1)^ |
| Species _Children_ | -0.956 | 0.434 | -1.943 | -0.054 | ^(1)^ | ^(1)^ | ^(1)^ |
| Species _Pig_ | -2.023 | 0.532 | -3.460 | -1.139 |  |  |  |
| Action | -0.098 | 0.285 | -0.671 | 0.488 | ^(1)^ | ^(1)^ | ^(1)^ |
| Action Side _Right_ | -0.006 | 0.250 | -0.544 | 0.514 | 0.001 | 1 | 0.98 |
| Informant _Unreliable_ * Species _Children_ | 0.254 | 0.663 | -1.134 | 1.678 | 0.297 | 2 | 0.862 |
| Informant _Unreliable_ * Species _Pig_ | 0.343 | 0.659 | -0.926 | 1.704 |  |  |  |
| Informant _Unreliable_ * Action | 0.057 | 0.327 | -0.608 | 0.719 | 0.029 | 1 | 0.864 |
| Species _Children_ * Action | -0.749 | 0.329 | -1.558 | -0.169 | 5.401 | 2 | 0.067 |
| Species _Pig_ * Action | -0.293 | 0.349 | -1.132 | 0.435 |  |  |  |

^(1)^ not shown due to very limited interpretability

**Table S4C**
Results of the final reduced GLMM investigating the probability to follow the evidence in all first trials of each informant per action (estimates, together with standard errors, confidence limits, significance tests). Model reduced by removing any interaction involving informant role.

|  | **Estimate** | **SE** | **lwr CI** | **upr CI** | **Chisq** | **Df** | **P** |
| --- | --- | --- | --- | --- | --- | --- | --- |
| (Intercept) | 1.809 | 0.293 | 1.367 | 2.621 | ^(1)^ | ^(1)^ | ^(1)^ |
| Informant _Unreliable_ | 0.300 | 0.228 | -0.166 | 0.836 | 1.738 | 1 | 0.187 |
| Species _Children_ | -0.883 | 0.290 | -1.626 | -0.305 | 16.67 | 2 | <0.001 |
| Species _Pig_ | -1.878 | 0.418 | -2.982 | -1.103 |  |  |  |
| Action | -0.438 | 0.138 | -0.808 | -0.172 | 7.845 | 1 | 0.005 |
| Action Side _Right_ | -0.002 | 0.247 | -0.490 | 0.521 | 0 | 1 | 0.993 |

^(1)^ not shown due to very limited interpretability

### Choice Latencies

**Table S5**Results of the full LMM investigating choice latencies (estimates, together with standard errors, confidence limits, significance tests, as well as minimum and maximum of estimates obtained after dropping individuals one at a time).

|  | **Estimate** | **SE** | **lwr CI** | **upr CI** | **F** | **Df** | **P** | **min** | **max** |
| --- | --- | --- | --- | --- | --- | --- | --- | --- | --- |
| Full null model comparison |  | | | | X^2^ = 19.484 | 11 | 0.053 |  | |
| (Intercept) | 1.238 | 0.085 | 1.066 | 1.407 | ^(1)^ | ^(1)^ | ^(1)^ | 1.190 | 1.285 |
| Informant _Unreliable_ | -0.006 | 0.048 | -0.101 | 0.089 | ^(1)^ | ^(1)^ | ^(1)^ | -0.044 | 0.012 |
| Species _Children_ | 0.330 | 0.117 | 0.105 | 0.576 | ^(1)^ | ^(1)^ | ^(1)^ | 0.239 | 0.370 |
| Species _Pig_ | 0.821 | 0.132 | 0.551 | 1.088 |  |  |  | 0.775 | 0.914 |
| Action | 0.024 | 0.062 | -0.098 | 0.147 | ^(1)^ | ^(1)^ | ^(1)^ | -0.010 | 0.056 |
| Action Side _Right_ | 0.002 | 0.026 | -0.047 | 0.053 | 0.007 | 1 | 0.937 | -0.013 | 0.018 |
| Peeking _Peeked_ | 0.801 | 0.102 | 0.601 | 1.019 | 50.877 | 1 | 0.002 | 0.716 | 0.839 |
| Informant _Unreliable_ * Species _Children_ | 0.097 | 0.067 | -0.045 | 0.231 | ^(1)^ | ^(1)^ | ^(1)^ | 0.056 | 0.117 |
| Informant _Unreliable_ * Species _Pig_ | -0.010 | 0.068 | -0.161 | 0.135 |  |  |  | -0.028 | 0.025 |
| Informant _Unreliable_ * Action | -0.010 | 0.063 | -0.142 | 0.115 | ^(1)^ | ^(1)^ | ^(1)^ | -0.056 | 0.014 |
| Species _Children_ * Action | 0.136 | 0.110 | -0.083 | 0.347 | ^(1)^ | ^(1)^ | ^(1)^ | 0.074 | 0.218 |
| Species _Pig_ * Action | -0.010 | 0.104 | -0.226 | 0.201 |  |  |  | -0.053 | 0.055 |
| Informant _Unreliable_ * Species _Children_ * Action | 0.094 | 0.113 | -0.123 | 0.319 | 0.431 | 2 | 0.673 | 0.017 | 0.171 |
| Informant _Unreliable_ * Species _Pig_ * Action | -0.026 | 0.103 | -0.234 | 0.187 |  |  |  | -0.063 | 0.043 |

^(1)^ not shown due to very limited interpretability

### Choice Latencies: First Trials

**Table S6A**
Results of the full LMM investigating choice latencies in the first trials (estimates, together with standard errors, confidence limits, significance tests, as well as minimum and maximum of estimates obtained after dropping individuals one at a time).

|  | **Estimate** | **SE** | **lwr CI** | **upr CI** | **F** | **Df** | **P** | **min** | **max** |
| --- | --- | --- | --- | --- | --- | --- | --- | --- | --- |
| Full null model comparison |  | | | | X^2^ = 36.898 | 11 | <0.001 |  | |
| (Intercept) | 1.065 | 0.148 | 0.797 | 1.336 | ^(1)^ | ^(1)^ | ^(1)^ | 0.884 | 1.128 |
| Informant _Unreliable_ | 0.146 | 0.178 | -0.211 | 0.484 | ^(1)^ | ^(1)^ | ^(1)^ | 0.062 | 0.295 |
| Species _Children_ | 0.336 | 0.210 | -0.083 | 0.756 | ^(1)^ | ^(1)^ | ^(1)^ | 0.257 | 0.463 |
| Species _Pig_ | 0.687 | 0.236 | 0.239 | 1.154 |  |  |  | 0.587 | 0.896 |
| Action | 0.068 | 0.072 | -0.064 | 0.201 | ^(1)^ | ^(1)^ | ^(1)^ | 0.028 | 0.135 |
| Action Side _Right_ | 0.001 | 0.049 | -0.093 | 0.098 | 0.001 | 1 | 0.982 | -0.034 | 0.025 |
| Peeking _Peeked_ | 0.793 | 0.113 | 0.558 | 1.010 | 42.575 | 1 | 0.002 | 0.725 | 0.888 |
| Informant _Unreliable_ * Species _Children_ | -0.206 | 0.261 | -0.710 | 0.308 | ^(1)^ | ^(1)^ | ^(1)^ | -0.303 | -0.120 |
| Informant _Unreliable_ * Species _Pig_ | 0.190 | 0.299 | -0.397 | 0.764 |  |  |  | -0.039 | 0.338 |
| Informant _Unreliable_ * Action | -0.142 | 0.086 | -0.304 | 0.032 | ^(1)^ | ^(1)^ | ^(1)^ | -0.213 | -0.090 |
| Species _Children_ * Action | 0.017 | 0.104 | -0.190 | 0.213 | ^(1)^ | ^(1)^ | ^(1)^ | -0.034 | 0.073 |
| Species _Pig_ * Action | 0.048 | 0.117 | -0.184 | 0.277 |  |  |  | -0.014 | 0.083 |
| Informant _Unreliable_ * Species _Children_ * Action | 0.200 | 0.127 | -0.047 | 0.450 | 1.472 | 2 | 0.232 | 0.161 | 0.262 |
| Informant _Unreliable_ * Species _Pig_ * Action | -0.008 | 0.143 | -0.294 | 0.259 |  |  |  | -0.063 | 0.092 |

^(1)^ not shown due to very limited interpretability


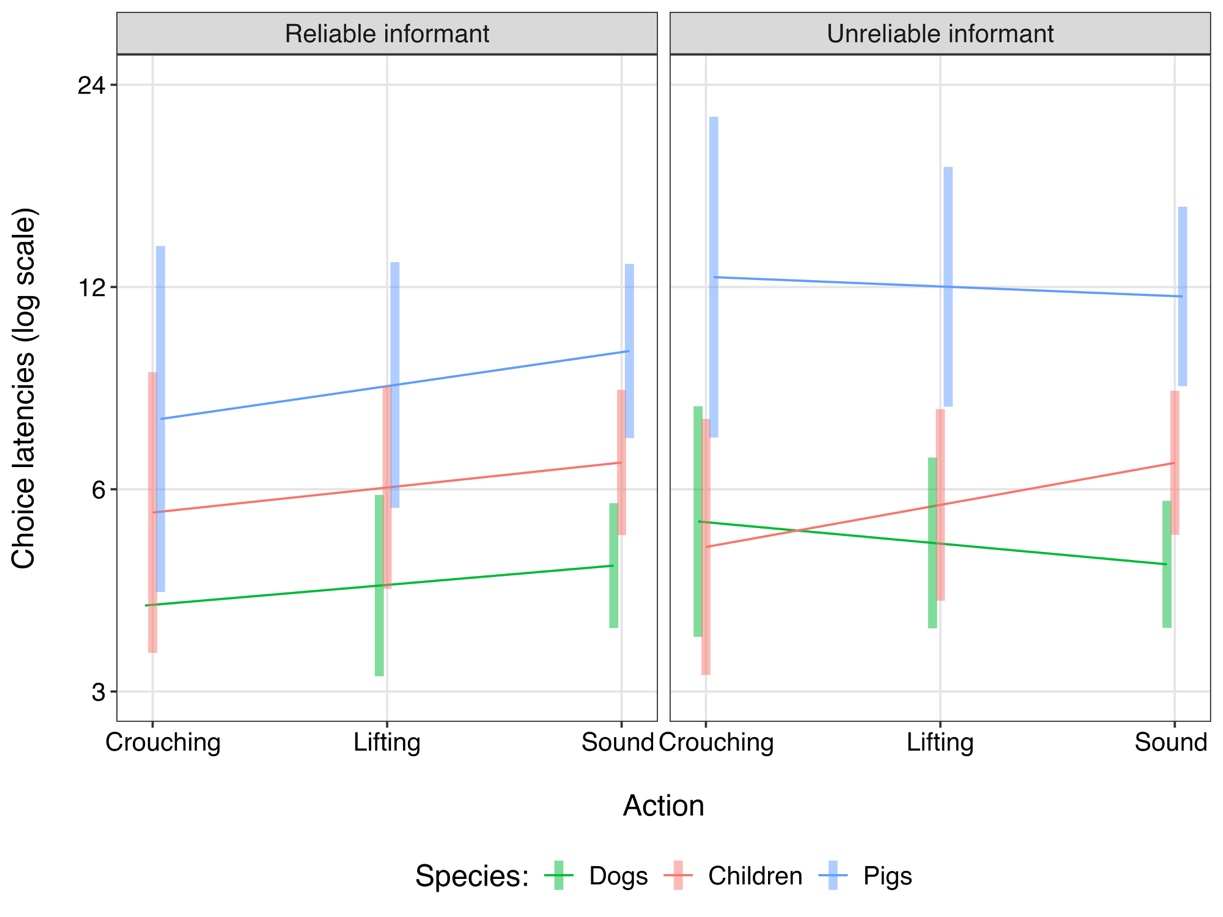
**Figure S4**Predicted change in choice latencies over actions per species and informant, in the first trials of each action and informant.

*Note.* Confidence level used: 0.95. Results are averaged over levels of action side and peeking. The depicted three-way interaction is not significant.

**Table S6B**
Results of the reduced LMM investigating choice latencies in the first trials (estimates, together with standard errors, confidence limits, significance tests, as well as minimum and maximum of estimates obtained after dropping individuals one at a time). Model reduced by removing the 3-way interaction of informant role, species and action.

|  | **Estimate** | **SE** | **lwr CI** | **upr CI** | **F** | **Df** | **P** |
| --- | --- | --- | --- | --- | --- | --- | --- |
| (Intercept) | 1.137 | 0.132 | 0.860 | 1.421 | ^(1)^ | ^(1)^ | ^(1)^ |
| Informant _Unreliable_ | 0.010 | 0.128 | -0.243 | 0.279 | ^(1)^ | ^(1)^ | ^(1)^ |
| Species _Children_ | 0.126 | 0.164 | -0.192 | 0.460 | ^(1)^ | ^(1)^ | ^(1)^ |
| Species _Pig_ | 0.701 | 0.184 | 0.343 | 1.069 |  |  |  |
| Action | 0.025 | 0.061 | -0.090 | 0.149 | ^(1)^ | ^(1)^ | ^(1)^ |
| Action Side _Right_ | 0.004 | 0.050 | -0.094 | 0.106 | 0.007 | 1 | 0.936 |
| Peeking _Peeked_ | 0.785 | 0.125 | 0.541 | 1.050 | 39.268 | 1 | 0.002 |
| Informant _Unreliable_ * Species _Children_ | 0.176 | 0.097 | -0.037 | 0.368 | 2.032 | 2 | 0.133 |
| Informant _Unreliable_ * Species _Pig_ | 0.174 | 0.113 | -0.052 | 0.392 |  |  |  |
| Informant _Unreliable_ * Action | -0.070 | 0.057 | -0.190 | 0.035 | 1.482 | 1 | 0.227 |
| Species _Children_ * Action | 0.138 | 0.071 | 0.001 | 0.280 | 1.909 | 2 | 0.153 |
| Species _Pig_ * Action | 0.042 | 0.080 | -0.116 | 0.190 |  |  |  |

^(1)^ not shown due to very limited interpretability

**Table S6C**
Results of the reduced LMM investigating choice latencies in the first trials (estimates, together with standard errors, confidence limits, significance tests, as well as minimum and maximum of estimates obtained after dropping individuals one at a time). Model reduced by removing any interaction.

|  | **Estimate** | **SE** | **lwr CI** | **upr CI** | **F** | **Df** | **P** |
| --- | --- | --- | --- | --- | --- | --- | --- |
| (Intercept) | 1.035 | 0.095 | 0.863 | 1.222 | ^(1)^ | ^(1)^ | ^(1)^ |
| Informant _Unreliable_ | -0.014 | 0.049 | -0.117 | 0.081 | 0.077 | 1 | 0.790 |
| Species _Children_ | 0.463 | 0.096 | 0.262 | 0.653 | 35.898 | 2 | <0.001 |
| Species _Pig_ | 0.877 | 0.106 | 0.671 | 1.095 |  |  |  |
| Action | 0.044 | 0.031 | -0.018 | 0.107 | 1.959 | 1 | 0.164 |
| Action Side _Right_ | 0.012 | 0.049 | -0.087 | 0.100 | 0.058 | 1 | 0.810 |
| Peeking _Peeked_ | 0.827 | 0.131 | 0.572 | 1.098 | 39.912 | 1 | 0.002 |

^(1)^ not shown due to very limited interpretability

**
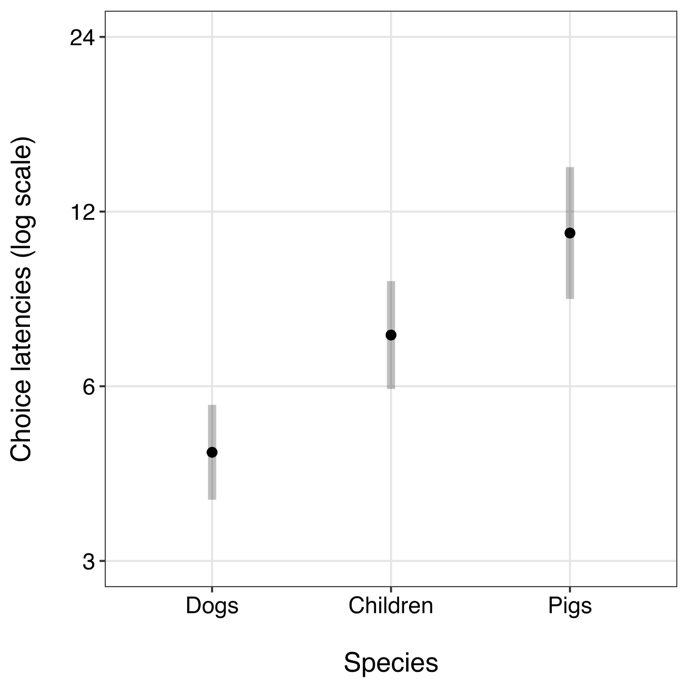
Figure S5**Predicted choice latencies for each species in the first trials in each action with each informant.

*Note.* Confidence intervals are given at the 95% level. Results are averaged over levels of action side and peeking.

## Transfer Phase

**Table S7**
Results of the full GLMM investigating the probability to choose the Reliable over the Unreliable informant in the transfer tasks (estimates, together with standard errors, confidence limits, significance tests, as well as minimum and maximum of estimates obtained after dropping individuals one at a time).

|  | **Estimate** | **SE** | **lwr CI** | **upr CI** | **Chisq** | **Df** | **P** | **min** | **max** |
| --- | --- | --- | --- | --- | --- | --- | --- | --- | --- |
| Full null model comparison |  | | | | 0.778 | 5 | 0.978 |  | |
| (Intercept) | -0.098 | 0.251 | -0.630 | 0.443 | ^(1)^ | ^(1)^ | ^(1)^ | -0.189 | 0.021 |
| Species _Children_ | 0.139 | 0.321 | -0.484 | 0.798 | ^(1)^ | ^(1)^ | ^(1)^ | -0.032 | 0.345 |
| Species _Pig_ | 0.036 | 0.284 | -0.505 | 0.669 |  |  |  | -0.399 | 0.300 |
| Task | 0.108 | 0.135 | -0.165 | 0.406 | ^(1)^ | ^(1)^ | ^(1)^ | 0.039 | 0.167 |
| Trial within Task | 0.134 | 0.120 | -0.103 | 0.391 | 1.275 | 1 | 0.259 | 0.079 | 0.228 |
| Side of Reliable _Right_ | -0.233 | 0.216 | -0.671 | 0.201 | 1.207 | 1 | 0.272 | -0.389 | -0.100 |
| Species _Children_ * Task | -0.117 | 0.222 | -0.563 | 0.314 | 0.280 | 2 | 0.869 | -0.179 | -0.039 |
| Species _Pig_ * Task | -0.028 | 0.207 | -0.447 | 0.360 |  |  |  | -0.153 | 0.216 |

^(1)^ not shown due to very limited interpretability


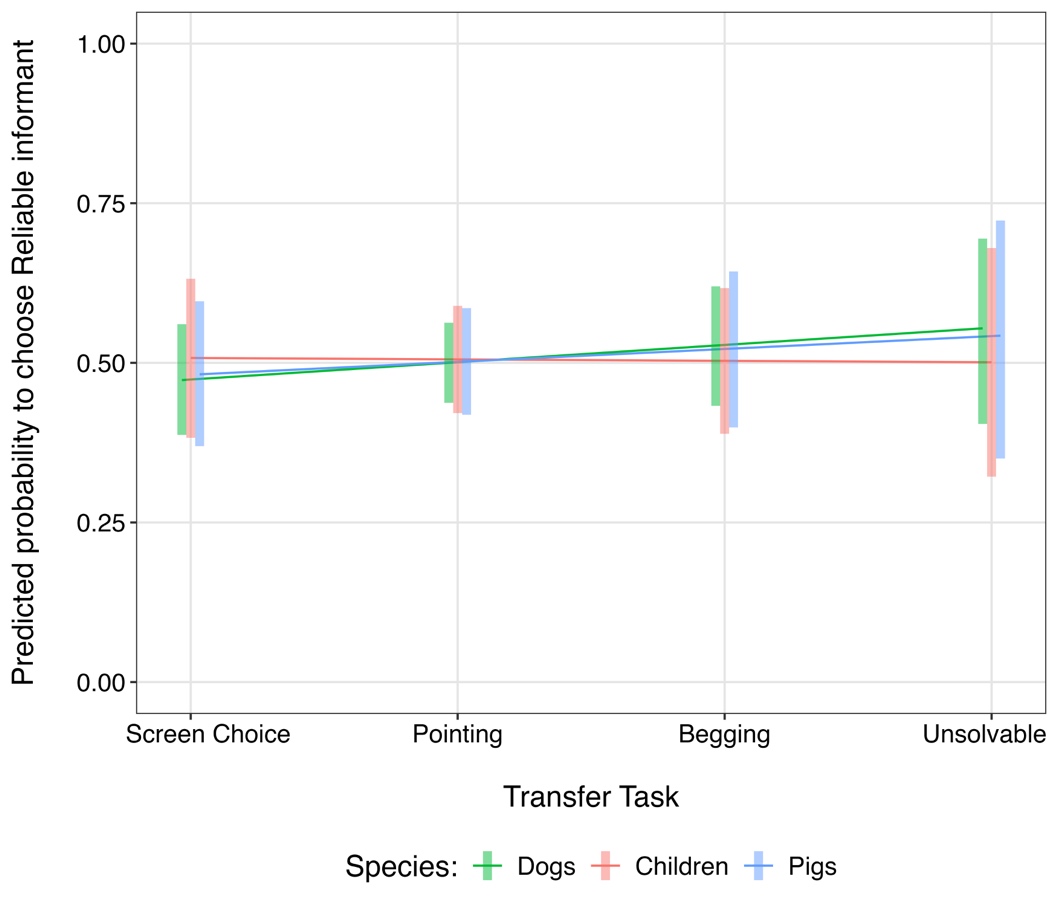
**Figure S6**Predicted probability to choose the Reliable over the Unreliable informant per species, over the four transfer tasks.

*Note.* Confidence levels: 0.95. Results are averaged over trial within task and Reliable informant side. The depicted interaction is not significant.
